# Synthesis and characterization of dendritic compounds containing nitrogen: monomer precursors in the construction of biomimetic membranes

**DOI:** 10.1038/s41598-022-05747-1

**Published:** 2022-02-02

**Authors:** Jordi Guardià, Alireza Zare, John Eleeza, Marta Giamberini, José Antonio Reina, Xavier Montané

**Affiliations:** 1 Department of Analytical Chemistry and Organic Chemistry, Universitat Rovira i Virgili (URV), C/Marcel·lí Domingo 1, 43007 Tarragona, Spain; 2 Department of Chemical Engineering, Universitat Rovira i Virgili (URV), Av. Països Catalans 26, 43007 Tarragona, Spain

**Keywords:** Self-assembly, Liquid crystals, Synthetic chemistry methodology

## Abstract

In this article, we synthesized a novel dendritic 2-oxazoline, 2-(3,4,5-tris(4-dodecyloxybenzyloxy)phenyl)-4,5-dihydro-1,3-oxazole), and its amide precursor *N*-(2-hydroxyethyl)-3,4,5-tris(4-dodecyloxybenzyloxy)benzamide. Of the distinct synthetic routes explored, it was established that the direct amidation of esters with sodium methoxide followed by the dehydrative cyclisation with 2,3-dichloro-5,6-dicyano-1,4-benzoquinone as oxidizing agent and triphenylphosphine was the most efficient route to synthesize the dendritic 2-oxazoline. Besides, *N*-(2-hydroxyethyl)-3,4,5-tris(4-dodecyloxybenzyloxy)benzamide exhibited a monotropic columnar mesophase, whilst the dendritic 2-oxazoline does not exhibited a liquid crystalline mesophase. At the end, the first attempts to polymerize the 2-oxazoline monomer via cationic ring opening polymerization showed promising results. Therefore, the dendritic 2-oxazoline could be used as a mesogenic monomer in the synthesis of side-chain liquid-crystalline polyoxazolines that might self-assembly into columnar structures.

## Introduction

4,5-Dihydro-1,3-oxazoles, more commonly known as 2-oxazolines or Δ^2^-1,3-oxazolines, are five-membered cyclic imino ethers which were synthesised for the first time in 1884^1^, although their structure was not clearly elucidated until 5 years later by Gabriel^2^. Since then, distinct synthetic routes have been described for the synthesis of these heterocyclic organic compounds, being direct synthesis from carboxylic acids or their derivatives (esters or acid halides) and the cyclisation of β-hydroxyamides with different dehydrating agents the most typically reported pathways^3,4^.

Different applications have been reported for 2-oxazolines: they have been utilised as ligands in asymmetric catalysis^5,6^, as synthetic intermediates^7^, as structural components of natural products or as protecting groups for carboxylic acid functionalities^8^. Furthermore, these cyclic organic compounds are principally employed to build-up substituted poly(2-oxazoline)s (PAOx) with a well-defined structure by living cationic ring-opening polymerization (CROP)^3,9–11^. In these cases, the substituent of the 2-oxazoline cycle determines the properties of the obtained poly(2-oxazoline)s. Precisely, the similar chemical structure of the resulting poly(2-oxazoline)s to natural polypeptides confers to these polymers an excellent biocompatibility, which together with their low viscosity and high stability make this kind of polymeric materials an ideal candidate in biomedical applications. As proof of these exceptional properties, Moreadith and co-workers reported recently a drug loaded PAOx that has been tested as a therapeutic agent in the Phase 1 clinical trials of Parkinson’s disease^12^.

Side-chain liquid-crystalline polymers (SCLCPs) have aroused a great interest due to its self-assembling ability, which gives them unique optical, electrical and mechanical properties^13^. Therefore, the well-ordered SCLCPs have been used in a vast range of applications in many fields such as optical data storage^14^, non-linear optics^15^, optical compensators^16^, separation membranes^17,18^, solid polymer electrolytes^19,20^ or electronic devices^21,22^.

According to that, the synthesis of the first liquid-crystalline poly(2-oxazoline)s via CROP of 2-substituted 2-oxazolines, which contain a side-chain mesogenic group, was presented by the research group of Virgil Percec in 1987^23^. Since then, this group conducted a wide investigation on the self-assembly of several minidendritic 2-oxazolines and the subsequent self-organisation of the dendronized poly(ethyleneimine)s into LC phases via self-assembly process^24–27^.

Furthermore, Kim and co-workers reported the synthesis of a family of side-chain liquid-crystalline poly(2-oxazoline)s with different contents of cyanobiphenyl-based mesogenic groups^28^. Moreover, they grafted distinct mesogens that differ in the methylene units of the spacer length (2, 4, 6 and 10 methylene units, respectively) to a partially hydrolysed poly(2-methyl-2-oxazoline) via Steglich esterification. They proved that at least a modification degree equal to 27% of cyanobiphenyl mesogenic group is required in the resulting poly(2-oxazoline)s to detect a liquid-crystalline mesophase by DSC (differential scanning calorimetry) or POM (polarized optical microscopy). Furthermore, the LC phase range of the poly(2-oxazoline)s, which exhibit smectic or nematic LC textures, is correlated with the length of the spacer moiety of the mesogen.

In the last decades, our research group has focused on the design and synthesis of dendronized liquid-crystalline polyethers and polyamines by grafting dendritic side groups into these polymers, which induce their self-assembly into columnar structures, thus resulting in the formation of an inner channel, which contains polar ether or amine linkages, which allow it to work as an ion channel^29–31^. Membranes prepared using the previously synthesized low molecular weight side-chain liquid-crystalline polyamines exhibited a remarkable proton transport, despite their poor mechanical properties and brittleness^32,33^. To minimize these drawbacks, in this paper the synthesis of higher molecular weight side-chain liquid crystalline polyamines was tackled by the polymerization of dendritic monomers. It must be mentioned that the synthesis and characterization of a dendritic liquid-crystalline aziridine was reported by us^34^, which exhibited a monotropic columnar LC phase on cooling. Even so, attempts to polymerize it did not provide satisfactory results^34^.

Thus, in this work, we describe the synthesis and characterization of a novel dendritic 2-oxazoline monomer 2-(3,4,5-tris(4-dodecyloxybenzyloxy)phenyl)-4,5-dihydro-1,3-oxazole) (TAPOx) and its amide precursor *N*-(2-hydroxyethyl)-3,4,5-tris(4-dodecyloxybenzyloxy)benzamide (TAPAm). The 2-oxazoline monomer can be subsequently polymerized in a controlled manner taking advantage of the “living” character of CROP of this type of monomers to obtain side-chain liquid-crystalline acyl-substituted poly(ethyleneimine)s, that can be a promising candidate in the preparation of membranes that allow the transport of cations thanks to the presence of nitrogen atoms in their inner channel. Moreover, the nature of the CROP would let us to obtain high molecular weight SCLCPs, which will enable the preparation of membranes that should exhibit improved mechanical properties than the cation exchanges membranes tested in polymer electrolyte fuel cells with the previously synthesized side-chain liquid-crystalline polyethers and polyamines.

## Experimental

### Materials

1-Ethyl-3-(3-dimethylaminopropyl)carbodiimide (EDC, > 97%), 4-dimethylaminopyridine (DMAP, ≥ 99%), ethanolamine (≥ 99%), sodium methoxide (NaOMe, solution 0.5 M in methanol), n-butyllithium (n-BuLi, solution 2.5 M in hexanes), thionyl chloride (SOCl_2_, ≥ 99%), anhydrous lanthanum triflate (LaTf_3_, 99.999%), anhydrous lanthanum chloride (LaCl_3_, 99.9%), methyl tosylate (MeOTs, 98%), anhydrous benzotrifluoride (≥ 99%) and dodecylamine (98%) were purchased from Sigma Aldrich. 1,8-Diazabicyclo[5.4.0]undec-7-ene (DBU, 99%) and 2,3-dichloro-5,6-dicyano-1,4-benzoquinone (DDQ, 98%) were supplied by Alfa Aesar. *N,N′*-Dicyclohexylcarbodiimide (DCC, ≥ 99%) was purchased from Fluka and triphenylphosphine (PPh_3_, 99%) was supplied by Acros Organics. The other solvents were supplied by Scharlab. Furthermore, toluene, dichloromethane (DCM), dimethylformamide (DMF) and ethanolamine were dried before use according to literature^35^.

### Synthesis of the tapered mesogenic precursors

The synthesis of methyl 3,4,5-tris[*p*-(n-dodecan-1-yloxy)benzyloxy]benzoate (TAPEs) was performed following a slight modification of a reported procedure that involved easier workup and higher yield^36^:

*p*-(n-dodecan-1-yloxy)benzyl chloride (36.1 g, 0.12 mol), methyl 3,4,5-trihydroxybenzoate (7.1 g, 0.04 mol), powdered potassium carbonate (48.1 g, 0.35 mol) and dry DMF (400 mL) were added into a twin-neck round bottom flask. The mixture was stirred and heated at 60 °C during 4 h under argon atmosphere. The reaction was monitored by thin layer chromatography (TLC) using toluene as an eluent. After complete conversion of *p*-(n-dodecan-1-yloxy)benzyl chloride, the reaction was poured to 1.5 L of ice water, filtered, and recrystalized twice in acetone. Finally, the product was dried under vacuum at room temperature to yield 30.1 g (77%) of a white solid.

The synthesis of 3,4,5-tris-[4-(n-dodecan-1-yloxy)benzyloxy]benzoic acid (TAPAc) was carried out as described elsewhere^31^.

### Synthesis of *N*-(2-hydroxyethyl)-3,4,5-tris(4-dodecyloxybenzyloxy)benzamide (TAPAm)

#### Direct amidation method using EDC as coupling agent

In a round-bottomed flask, 0.50 g of TAPAc (0.50 mmol) were dissolved into 10 mL of chloroform. The solution was stirred at 0 °C in an ice-water bath for 15 min. Then, DMAP (49.2 mg, 0.40 mmol) was added, and the solution was kept under stirring at 0 °C during 15 min more. After that, EDC (97.3 mg, 0.50 mmol) was added, leaving the magnetic stirring for additionally 15 min at the same temperature. At this moment, ethanolamine (0.09 mL, 1.49 mmol) was added dropwise. When the addition of ethanolamine was completed, the reaction mixture was warmed to room temperature and kept under stirring. The conversion of TAPAc was monitored by TLC using n-hexane/ethyl acetate as mixture of eluents (1:2). After complete conversion of TAPAc was observed by TLC (27 h), the crude mixture was first extracted twice with water and then once with brine. The organic layer was dried over anhydrous MgSO_4_ and vacuum evaporated. Finally, the obtained solid was purified by flash column chromatography (gradient of n-hexane/ethyl acetate as mixture of eluents; starting proportion = 2:1), obtaining pure TAPAm as a white powder (212.5 mg, 42%).

^1^H NMR [CDCl_3_, δ, ppm]: 7.23 (d, 4H, –O–**Ar–H**–CH_2_–O–Ar–CONH– from 2 and 6 positions of the lateral benzylic units), 7.16 (d, 2H, –O–**Ar–H**–CH_2_–O–Ar–CONH– from 2 and 6 positions of the central benzylic unit), 6.98 (s, 2H, **ArH**–CONH–), 6.80 (d, 4H, –O–**Ar–H**–CH_2_–O–Ar–CONH– from 3 and 5 positions of the lateral benzylic units), 6.67 (d, 2H, –O–**Ar–H**–CH_2_–O–Ar–CONH– from 3 and 5 positions of the central benzylic unit), 6.47 (t, 1H, –N**H**–), 4.93 (s, 4H, –**CH**_**2**_–O–Ar–CONH– in lateral benzylic units), 4.90 (s, 2H, –**CH**_**2**_–O–Ar–CONH– in central benzylic units), 3.86 (m, 4H, –Ar–O–**CH**_**2**_–(CH_2_)_10_–CH_3_ in lateral benzylic units), 3.83 (t, 2H, Ar–O–**CH**_**2**_–(CH_2_)_10_–CH_3_ in central benzylic units), 3.73 (t, 2H, –NH–CH_2_–**CH**_**2**_–OH), 3.50 (dt, 2H, –NH–CH_2_–CH_2_–OH), 1.70 (m, 6H, –Ar–O–CH_2_–**CH**_**2**_–(CH_2_)_9_–CH_3_), 1.37 (m, 6H, –Ar–O–CH_2_–CH_2_–**CH**_**2**_–(CH_2_)_8_–CH_3_), 1.20 (m, 48H, –Ar–O–CH_2_–CH_2_–CH_2_–**(CH**_**2**_**)**_**8**_–CH_3_), 0.81 (t, 9H, –Ar–O–CH_2_–(CH_2_)_10_–**CH**_**3**_).

^13^C NMR [CDCl_3_, δ, ppm]: 168.4 (–**C**ONH–), 159.2 (**ArC**–O–(CH_2_)_11_–CH_3_ in lateral and central benzylic units), 153.0 (**ArC** meta to –CONH–), 141.5 (**ArC** para to –CONH–), 130.4 (**ArC** meta to –O–(CH_2_)_11_–CH_3_ in central benzylic unit), 129.5 (**ArC**–CONH–), 129.4 (**ArC** meta to –O–(CH_2_)_11_–CH_3_ in lateral benzylic units), 128.6 (**ArC**–CH_2_–O–Ar–CONH–), 114.6 (**ArC** ortho to –O–(CH_2_)_11_–CH_3_ in lateral benzylic units), 114.2 (**ArC** ortho to –O–(CH_2_)_11_–CH_3_ in central benzylic unit), 107.3 (**ArC** ortho to –CONH–), 74.9 (–**CH**_**2**_–O–Ar–CONH– in central benzylic unit), 71.4 (–**CH**_**2**_–O–Ar–CONH– in lateral benzylic units), 68.2 (–**CH**_**2**_–(CH_2_)_10_–CH_3_ in central and lateral benzylic units), 62.5 (–NH–CH_2_–**CH**_**2**_–OH), 43.1 (–NH–**CH**_**2**_–CH_2_–OH), 32.1 (–**CH**_**2**_–CH_2_–CH_3_), 29.8–29.4 (**–(CH**_**2**_**)**_**6**_–CH_2_–CH_3_) and (–**CH**_**2**_–(CH_2_)_9_–CH_3_), 26.2 (–**CH**_**2**_–(CH_2_)_8_–CH_3_), 22.8 (–**CH**_**2**_–CH_3_), 14.2 (–**CH**_**3**_).

IR (cm^−1^): 3295 (ν (O–H)); 2950 (ν (C–H) in –CH_3_ (asymmetric)); 2917–2850 (ν (C–H) in –CH_3_ (symmetric) and –CH_2_– (asymmetric and symmetric)); 1614 (ν (C=O)); 1514 (ν (C=C–C aromatic)); 1333 (δ (O–H) in plane bend); 1243 (ν (=C–O–C) asymmetric); 1121 (ν (–C–O–H)); 817 (δ (C–H) *p*-disubstitution).

#### Direct amidation of TAPEs with ethanolamine

In a pear-shaped flask, 1.00 g of TAPEs (0.99 mmol) was dissolved in ethanolamine (1.98 g, 33.07 mmol). The crude of the reaction, which was monitored by TLC (n-hexane/ethyl acetate (1:2) as mixture of eluents), was vigorously stirred under reflux at 140 °C. After total conversion of TAPEs (15 h), the black solution was cooled to RT and 10 mL of CHCl_3_ were added. The organic solution was extracted three times with water and then once more with brine. After that, the organic layer was dried over anhydrous MgSO_4_ and vacuum evaporated. Finally, the product was recrystallized twice with ethanol, obtaining 456.6 mg of TAPAm (43%).

#### Synthesis of TAPAm using sodium methoxide. (NaHCO_3_) as catalyst

In a round bottomed flask, a mixture of sodium methoxide (0.64 mL, 0.31 mmol), methyl 3,4,5-tris[*p*-(n-dodecan-1-yloxy)benzyloxy]benzoate (3.17 g, 3.14 mmol), ethanolamine (0.31 mL, 5.03 mmol) and dry toluene (6 mL) was heated at 50 °C for 22 h under argon atmosphere. The reaction was monitored by TLC using n-hexane/ethyl acetate (1:2) as mixture of eluents. When the reaction was completed, the resulting mixture was quenched with an aqueous saturated NH_4_Cl solution (40 mL). After the extraction with ethyl acetate (× 3), the organic layer was separated, dried over anhydrous MgSO_4_ and the solvent vacuum evaporated. Finally, the obtained solid was purified by recrystallization in ethanol to obtain 2.98 g of TAPAm (92%).

### Synthesis of 2-(3,4,5-tris(4-dodecyloxybenzyloxy)phenyl)-2-oxazoline (TAPOx)

#### Synthesis of TAPOx using a PPh_3_-DDQ system

In a previously dried Schlenk tube, 258.3 mg of PPh_3_ (0.98 mmol) and 223.6 mg of DDQ (0.98 mmol) were dissolved in 5 mL of dry DCM under argon atmosphere. Then, the mixture was stirred for 3 min at room temperature. At this point, 680.2 mg of TAPAm (0.66 mmol) was then added. The reaction was monitored by TLC using n-hexane/ethyl acetate (1:2) as mixture of eluents. After 20 min, when a complete conversion of TAPAm was reached, the crude mixture was washed with an aqueous NaOH solution (5%, 40 mL). After that, the separated water layer was back-extracted with DCM (15 mL × 4). The two organic layers were combined and washed together with brine solution, dried over anhydrous MgSO_4_ and the solvent was vacuum evaporated. Finally, the brown solid was purified by flash column chromatography using n-hexane/ethyl acetate (2:1) as mixture of eluents, giving the corresponding product as white solid (570.6 mg, 85%).

^1^H NMR [CDCl_3_, δ, ppm]: 7.25 (d, 4H, –O–**Ar–H**–CH_2_–O–Ar–C=N– from 2 and 6 positions of the lateral benzylic units), 7.21 (s, 2H, **Ar–H**–C=N–), 7.17 (d, 2H, –O–**Ar–H**–CH_2_–O–Ar–C=N– from 2 and 6 positions of the central benzylic unit), 6.83 (d, 4H, –O–**Ar–H**–CH_2_–O–Ar–C=N– from 3 and 5 positions of the lateral benzylic units), 6.67 (d, 2H, –O–**Ar–H**–CH_2_–O–Ar–C=N– from 3 and 5 positions of the central benzylic unit), 4.96 (s, 4H, –**CH**_**2**_–O–Ar–C=N– in lateral benzylic units), 4.91 (s, 2H, –**CH**_**2**_–O–Ar–C=N– in central benzylic unit), 4.34 (t, 2H, –C=N–CH_2_–**CH**_**2**_–O–), 3.97 (t, 2H, –C=N–**CH**_**2**_–CH_2_–O–), 3.89 (t, 4H, –Ar–O–**CH**_**2**_–(CH_2_)_10_–CH_3_ in lateral benzylic units), 3.84 (t, 2H, Ar–O–**CH**_**2**_–(CH_2_)_10_–CH_3_ in central benzylic units), 1.70 (m, 6H, –Ar–O–CH_2_–**CH**_**2**_–(CH_2_)_9_–CH_3_), 1.38 (m, 6H, –Ar–O–CH_2_–CH_2_–**CH**_**2**_–(CH_2_)_8_–CH_3_), 1.20 (m, 48H, –Ar–O–CH_2_–CH_2_–CH_2_–(**CH**_**2**_**)**_**8**_–CH_3_), 0.81 (t, 9H, –Ar–O–CH_2_–(CH_2_)_10_–**CH**_**3**_).

^13^C NMR [CDCl_3_, δ, ppm]: 164.7 (–**C**=N–), 159.1 (**ArC**–O–(CH_2_)_11_–CH_3_ in lateral and central benzylic units), 152.9 (**ArC** meta to –C=N–), 141.2 (**ArC** para to –C=N–), 130.4 (**ArC** meta to –O–(CH_2_)_11_–CH_3_ in central benzylic unit), 129.7 (**ArC**–CH_2_–O–Ar–C=N– in central benzylic unit), 129.4 (**ArC** meta to –O–(CH_2_)_11_–CH_3_ in lateral benzylic units), 128.9 (**ArC**–CH_2_–O–Ar–C=N– in lateral benzylic units), 122.9 (**ArC**–C=N–), 114.6 (**ArC** ortho to –O–(CH_2_)_11_–CH_3_ in lateral benzylic units), 114.2 (**ArC** ortho to –O–(CH_2_)_11_–CH_3_ in central benzylic units), 107.9 (**ArC** ortho to –C=N–), 74.8 (–**CH**_**2**_–O–Ar–C=N– in central benzylic unit) 71.2 (–**CH**_**2**_–O–Ar–C=N– in lateral benzylic units), 68.2 (–**CH**_**2**_–(CH_2_)_10_–CH_3_ in central and lateral benzylic units), 67.9 (–C=N–CH_2_–**CH**_**2**_–O–), 55.0 (–C=N–**CH**_**2**_–CH_2_–O–), 32.1 (–**CH**_**2**_–CH_2_–CH_3_), 29.8–29.5 (–**(CH**_**2**_**)**_**6**_–CH_2_–CH_3_) and (–**CH**_**2**_–(CH_2_)_9_–CH_3_), 26.2 (–**CH**_**2**_–(CH_2_)_8_–CH_3_), 22.8 (–**CH**_**2**_–CH_3_), 14.3 (–**CH**_**3**_).

IR (cm^−1^): 2950 (ν (C–H) in –CH_3_ (asymmetric)); 2917 – 2848 (ν (C–H) in –CH_3_ (symmetric) and –CH_2_– (asymmetric and symmetric)); 1643 (ν (C=N)); 1588 (ν (C=C–C aromatic)); 1514 (ν (C=C–C aromatic)); 1245 (ν (=C–O–C) asymmetric); 818 (δ (C–H) *p*-disubstitution).

### Preliminary polymerization studies

#### Bulk polymerization of TAPOx

In a previously flame-dried Schlenk tube, 505.0 mg of TAPOx monomer (0.49 mmol) were added. Then, the tube was immersed into a preheated oil bath at 130 °C, where TAPOx was stirred during 15 min under argon flow conditions. After that, 1% mol of methyl tosylate (0.91 mg, 4.9 · 10^–3^ mmol) were added and the reaction mixture was kept under argon until a complete conversion of TAPOx was detected by ^1^H NMR. At this point, an excess of dodecylamine (0.50 mL, 2.17 mmol) was added as terminating agent, maintaining the same temperature for 24 h, when the crude mixture was cooled to room temperature. Finally, the resulting mixture was dissolved in THF and the polyoxazoline was isolated by precipitation twice into cold methanol, obtaining 312.0 mg of a beige solid (62%).

### Characterization

#### Thermogravimetric analysis (TGA)

Thermal stability studies were carried out in ALU OXIDE crucibles of 70 µL (ME-24123) with a Mettler Toledo TGA2 thermobalance. All samples, weighing around 6–8 mg, were heated between 30 and 600 °C at a heating rate of 10 °C/min in N_2_ atmosphere with a flow rate of 50 cm^3^/min. The equipment was previously calibrated with indium (156.6 °C) and aluminium (660.3 °C) pearls.

#### Differential scanning calorimetry (DSC)

Calorimetric analyses were carried out on a Mettler DSC-821 instruments calibrated using indium (156.6 °C) and zinc (419.6 °C) pearls. Samples were placed in an aluminum standard crucible of 40 µL with pierced lids (between 4 – 6 mg of sample), which were analysed in N_2_ atmosphere (gas flow rate of 50 cm^3^/min). Heating and cooling rate of 10 °C/min has been always employed.

#### Polarized optical microscopy (POM)

LC mesophases were investigated by polarised optical microscopy (POM). The textures of the samples were observed with an Anxiolab Zeiss optical microscope equipped with a Linkam TP92 hot stage.

#### Nuclear magnetic resonance (NMR) spectroscopy

All synthesized compounds were characterized by ^1^H NMR and ^13^C NMR spectra, which were recorded in deuterated chloroform (CDCl_3_) with a Bruker Avance Neo 400 MHz spectrometer (^1^H—400 MHz; ^13^C—100.4 MHz) at room temperature. The chemical shifts were given in parts per million (ppm) from TMS (Tetramethylsilane) in ^1^H NMR spectra, while the central peak of the solvent was taken as reference in the case of ^13^C NMR spectra.

#### Fourier transform infrared (FT-IR) spectroscopy

FT-IR spectra were recorded on an FT/IR-6700 spectrophotometer from JASCO in the wavelength range of 4000–400 cm^−1^ with a resolution of 4 cm^−1^ in the absorbance mode. This device is equipped with an attenuated total reflection accessory (ATR) with thermal control and a diamond crystal (Golden Gate heated single reflection diamond ATR from Specac-Teknokroma). The spectra were recorded at room temperature from the solid-state pure compounds.

#### X-ray diffraction (XRD)

X-ray diffraction measurements (XRD) were made using a Bruker-AXS D8-Advance diffractometer with vertical θ-θ goniometer, incident- and diffracted-beam Soller slits of 2.5°, a fixed 0.5° receiving slit and an air-scattering knife on the sample surface. The angular 2θ range was between 2 and 40°. The data were collected with an angular step of 0.02° at a step/time of 0.5 s. Cukα radiation was obtained from a copper X-ray tube operated at 40 kV and 40 mA. Diffracted X-rays were detected with a PSD detector LynxEye-XE-T with an opening angle of 2.9°. The sample was placed inside an MTC-LOWTEMP chamber for in-situ temperature analysis.

## Results and discussion

The main goal of this work was the synthesis of a dendronized 2-oxazoline monomer bearing the tapered 3,4,5-tris[4-(n-dodecan-1-yloxy)benzyloxy)benzoate group, which will allow us to prepare LC poly(2-oxazoline)s by living CROP. Moreover, the thermal and mesomorphic characterization of the new mesogenic compounds was performed to determine their LC mesophases.

### Synthesis of 2-(3,4,5-tris(4-dodecyloxybenzyloxy)phenyl)-2-oxazoline (TAPOx)

Different synthetic routes have been explored in this work to synthesize 2-(3,4,5-tris(4-dodecyloxybenzyloxy)phenyl)-2-oxazoline (TAPOx), considering the number of involved stages. Besides, the following dendrons were used as starting materials:

- Methyl 3,4,5-tris[*p*-(n-dodecan-1-yloxy)benzyloxy]benzoate (TAPEs).
- 3,4,5-tris[*p*-(n-dodecan-1-yloxy)benzyloxy]benzoic acid (TAPAc).

**Figure 1:**
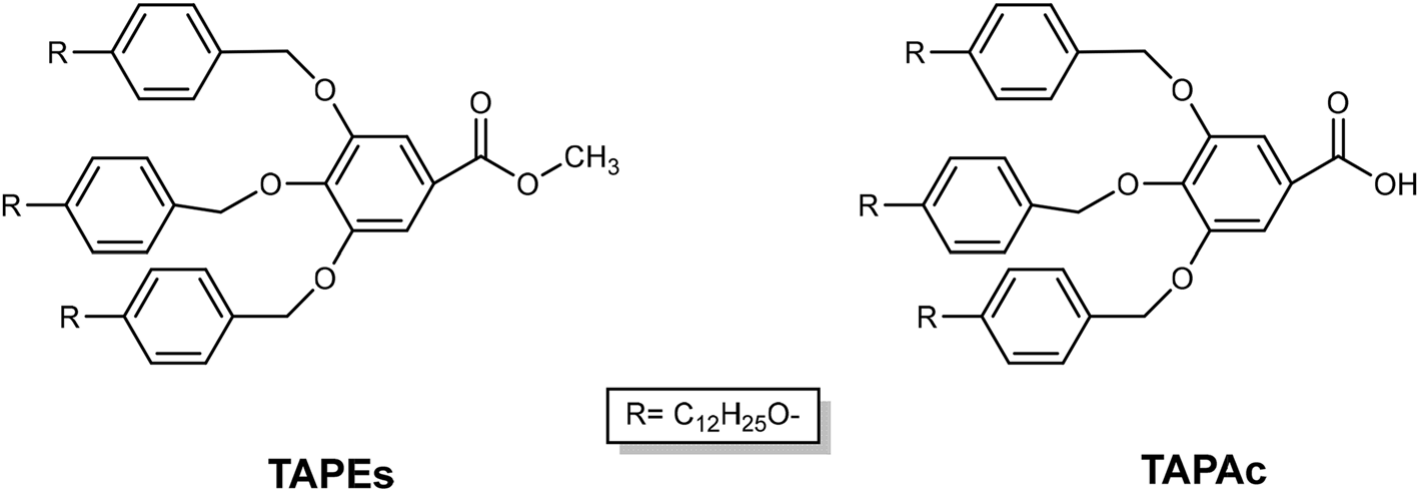
Chemical structure of: methyl 3,4,5-tris[*p*-(n-dodecan-1-yloxy) benzyloxy] benzoate (TAPEs) and 3,4,5-tris[*p*-(n-dodecan-1-yloxy) benzyloxy] benzoic acid (TAPAc).

The synthetic pathway of both dendrons was described in previous studies^31^ and in the experimental section of this paper. Their chemical structure is depicted in Fig. 1.

In this way, the first synthetic method selected comprised of two steps starting from TAPAc:

- The first step consisted of TAPAc amidation by means of DCC as coupling agent to obtain *N*-(2-hydroxyethyl)-3,4,5–tris(4-dodecyloxybenzyloxy)benzamide (TAPAm).
- The second step involve the cyclisation of TAPAm with thionyl chloride (SOCl_2_) to isolate the desired product (TAPOx).

Regarding the first step, the esterification of carboxylic acids using a carbodiimide as a promoter along with dimethylaminopyridine (DMAP) to activate an acid and favour the ester formation is one of the most common esterification methods employed due to the high number of advantages that this reaction shows: high versatility, mild conditions are usually required for the reaction to proceed (room temperature), which also facilitates its use with different types of substrates^37^. Moreover, it allows the obtention of esters from tertiary alcohols because the steric impediments do not affect this type of esterification. It was mentioned before that our group reported the chemical modification of polyethers and polyamines with the dendron 3,4,5-tris[*p*-(n-dodecan-1-yloxy)benzyloxy]benzoic acid (TAPAc) by means of Steglich esterification, obtaining side-chain liquid crystalline polyethers and polyamines^30–32^. The promoter that we used in all these studies was *N*,*N’*-dicyclohexylcarbodiimide (DCC)^31^.

Despite the advantages of Steglich esterification, this reaction presents some drawbacks: stoichiometric amounts or more of the carbodiimide are required, the yields are not always high and undesired *N*-acylureas are occasionally formed. These disadvantages may be minimized by the addition of strong acids like *p*-toluenesulfonic acid^38^, *N*-hydroxy derivatives^39^ or tertiary amines^40^, which suppress the formation of *N*-acylurea and favour the formation of the corresponding ester.

In this direction, the amidation of amines instead of alcohols to the corresponding amides took place with DCC as coupling agent following the same mechanism because amines present a more nucleophilic character. Therefore, the amidation of TAPAc with ethanolamine using the same conditions reported previously was carried out (Fig. S1 in Supplementary information). Although ethanolamine also contains a hydroxyl group, the amidation reaction will be favoured due to the chemoselectivity of the amine group present in ethanolamine versus the hydroxyl group. The dendronized amide was obtained in our case, even though a large amount of the *N*-acylurea side product was found mixed with TAPAm. Nonetheless, the complete removal of the dicyclohexylurea (DCU) was not possible by the purification methods employed in the work-up process of the crude mixture: filtration or flash column chromatography; obtaining always a highly impurified product.

To avoid this problem, DCC was replaced by a water soluble carbodiimide, 1-ethyl-3-(3-dimethylaminopropyl)carbodiimide (EDC). Despite EDC is an expensive reagent, the formed *N*-acylurea can be easily removed by aqueous extraction^39,41,42^. Besides, it was observed that the formation of the amide took place faster when EDC was used as coupling agent compared with the reaction using DCC (27 h vs*.* 4 days, respectively). Nonetheless, it was still necessary to purify the crude mixture by flash column chromatography.

Although amides can be obtained from carboxylic acids, we can take advantage of the greater reactivity of the ester group present in TAPEs to synthesize TAPAm^43,44^. In this way, Percec and co-workers reported a simple method for the amidation of dendronized esters into the corresponding amides by using a large excess of ethanolamine with yields higher than 80%^26,45^. So, the direct amidation of TAPEs with ethanolamine was also explored following the conditions reported by Percec group to increase the yield in the synthesis of TAPAm. Nevertheless, lower yields were obtained in our case (43%).

On the other hand, the addition of a catalyst facilitates the formation of amides from the corresponding esters under mild conditions with excellent yields. Different types of catalyst have been reported in literature, which exhibited an improved efficiency in the formation of amides: inorganic catalysts such as Sb(OEt)_3_^46^ or Zr(O^*t*^Bu)_4_-HOAt)^47^; or organic catalysts, including *N*-heterocyclic carbine^48^, DBU^49^ and triazabicyclo[4.4.0]dec-5-ene^50^. Furthermore, Yang and co-workers showed that the combination of different catalysts (1,2,4-triazole and DBU) accelerates the ester conversion to generate the corresponding amides at room temperature in high yields^51^. However, the preparation of many of these catalysts is quite complex, which implies that the catalytic activity of simpler and cheaper compounds is investigated. In this sense, sodium methoxide (NaOMe) is a well-known commercial compound that is widely used as catalyst in the production of biodiesel by transesterification of triglycerides with methanol^52^. When Ohshima et al. used catalytic amounts of sodium methoxide (≤ 10%) in the direct amidation of esters, they observed that the addition of this non-toxic catalyst results in the obtention of the corresponding amides under mild conditions, although this reaction requires the use of inert atmosphere to suppress the saponification of the starting esters^53^.

**Figure 2:**
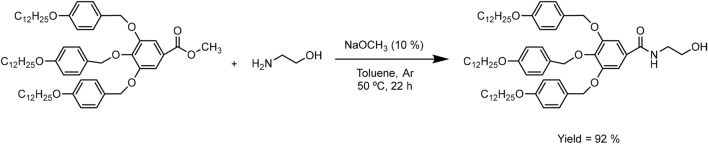
Synthesis of *N*-(2-hydroxyethyl)-3,4,5–tris(4-dodecyloxybenzyloxy) benzamide (TAPAm).

According to that, this procedure was followed in the amidation of TAPEs (Fig. 2). In our case, the addition of 10% mol of NaOMe not only simplified the work-up process of TAPAm, since flash column chromatography was replaced by a recrystallization in EtOH, but also caused an increase of more than double of the yield of the reaction with respect to the attempts reported before (92%).

Table 1 shows the different precursors, the solvent and the reaction conditions used in the chemical reactions tested in the synthesis of TAPAm, together with the obtained results.

**Table 1:** Starting materials, solvent, reaction conditions and results of the distinct chemical reactions carried out to obtain TAPAm.

Starting materials (mmols)	Solvent	Temperature (°C)	Time (h)	Yield (%)/comments
TAPAc (1.01) Ethanolamine (2.98) DCC (1.02) DMAP (0.83)	CHCl_3_	RT^a^	96	A mixture of TAPAm with *N,N’*-dicyclohexylurea was always isolated (TAPAm cannot be isolated alone from the final mixture)
TAPAc (0.50) Ethanolamine (1.49) EDC (0.50) DMAP (0.40)	CHCl_3_	RT^a^	27	42/The use of EDC allow the separation of TAPAm from water soluble *N,N’*-dicyclohexylureas However, a flash column chromatography is required to obtain pure TAPAm
TAPEs (0.99) Ethanolamine (33.07)	Ethanolamine	140	15	43/TAPAm was obtained after recrystallization with ethanol (× 2) Easy to scale-up
TAPEs (3.14) Ethanolamine (5.03) NaOCH_3_ (0.31)	Anhydrous toluene	50	22	92/TAPAm was obtained after recrystallization with ethanol (× 2) with a high yield Easy to scale-up

^a^RT, room temperature = 20 ± 3 °C.

To confirm its chemical structure, dendronized TAPAm precursor was characterized by NMR and FT-IR spectroscopy.

**Figure 3:**
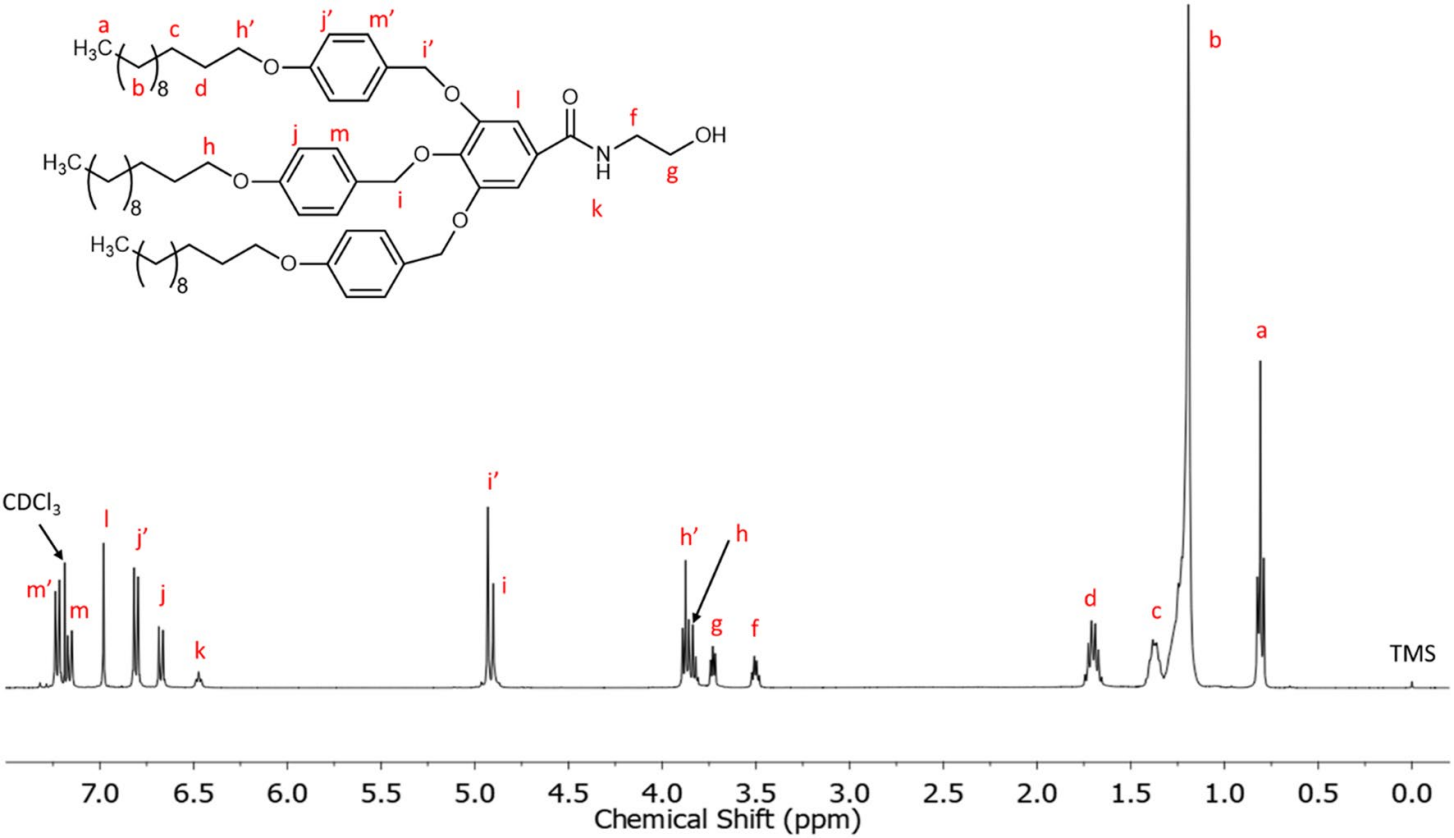
^1^H NMR spectrum in CDCl_3_ of TAPAm.

Figure 3 shows its ^1^H NMR spectrum, which was performed using CDCl_3_ as solvent. The aromatic region shows five signals at 7.23, 7.16, 6.98, 6.80 and 6.67 ppm. In comparison with previously reported characterization of methyl 3,4,5-tris[*p*-(n-dodecan-1-yloxy) benzyloxy] benzoate^54^, the signals at 7.23 and 7.16 ppm (6 H) can be assigned to the protons in *ortho* position to –CH_2_O– of lateral and central benzylic units, respectively. The signal at 6.98 ppm (2 H) corresponds to the protons of the benzoate group linked to the amide, whose multiplicity appears as a singlet. The other two signals at 6.80 and 6.67 ppm correspond to the protons in *metha* position to –CH_2_O– of the lateral and central benzylic units, respectively. Furthermore, a triplet at 6.47 ppm corresponds to the proton of the amide group (–NH–), which is coupled to the neighbouring methylene as observed in the recorded 2D NMR spectra (COSY and HSQC NMR spectra; Figs. S2 and S3 in Supplementary information). The two signals centred at 4.93 and 4.90 ppm are assigned to the benzylic methylenes of the dodecyloxybenzyloxy substituents. Moving upfield in the spectrum, two triplets centred at 3.86 and 3.83 ppm are attributed to methylene attached to the oxygen in the long alkyl chains in lateral and central benzylic units, respectively. As we move to lower chemical shifts, the presence of two characteristic signals at 3.73 and 3.50 ppm confirm that the amidation reaction took place because they are assigned to the *N*-(hydroxyethyl) moiety. Particularly, the triplet at 3.73 is assigned to the methylenic protons next to the hydroxylic group, while the signal at 3.50 ppm corresponds to the methylene unit contiguous to the amide group, since these protons are coupled to neighbouring methylene group and -NH proton as confirmed by 2D NMR experiments (COSY and HSQC; Figs. S2 and S3 in Supplementary information). Finally, the signals at 1.70, 1.37, 1.20 and 0.81 ppm of the high-field region can be assigned to the protons of the alkyl long chains of the dendron.

**Figure 4:**
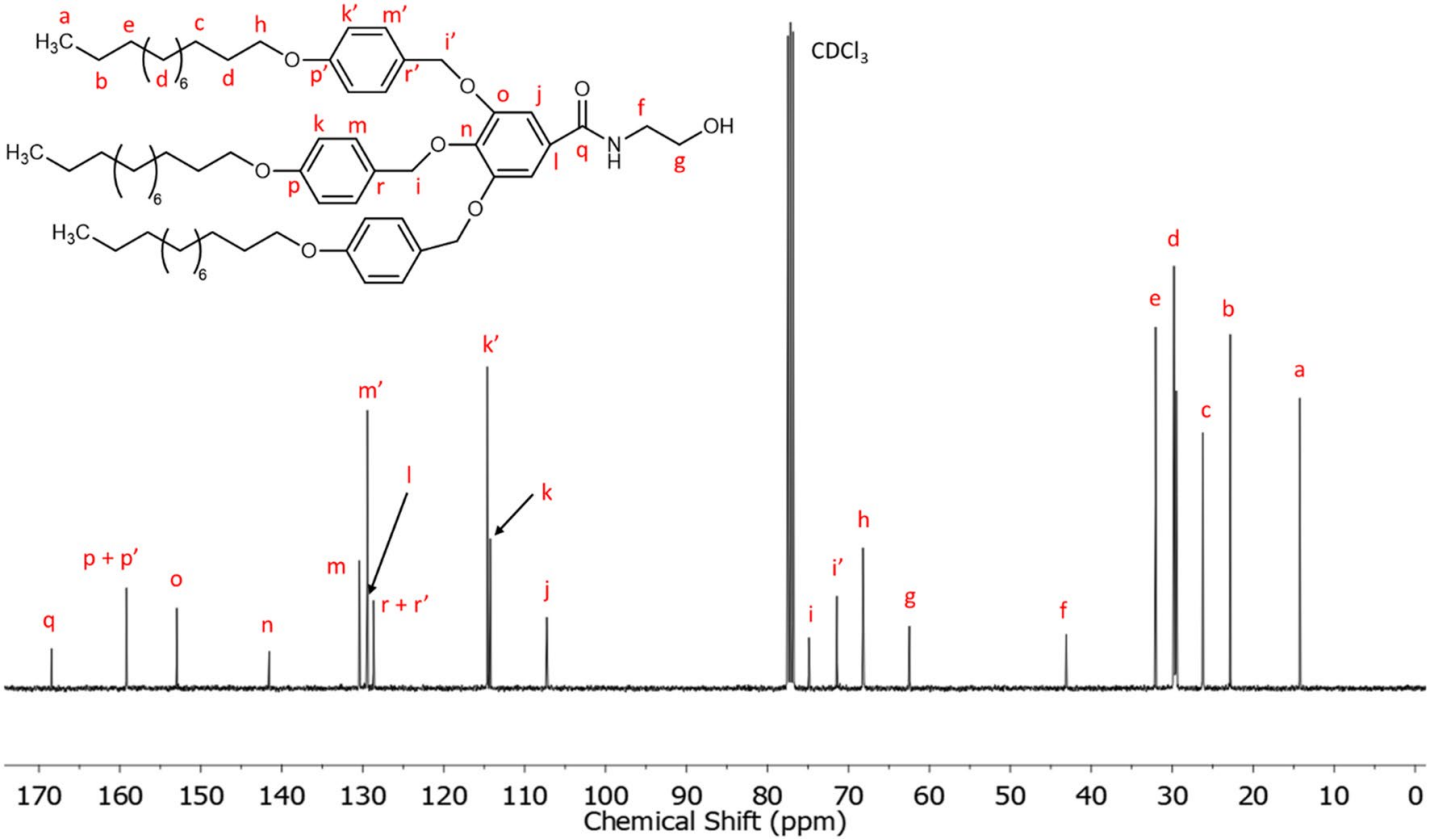
^13^C NMR spectrum in CDCl_3_ of TAPAm.

Figure 4 shows the ^13^C NMR of TAPAm with the corresponding assignments, taking the central peak of the solvent (CDCl_3_) as reference. The carbonyl carbon appears at 168.4 ppm, while the aromatic carbons appear between 159.2 and 107.3 ppm. The carbons that belong to the long aliphatic chain of the dendron (indexed with letters a – e) appear upfield in the spectrum (region between 32.1 and 14.2 ppm). The carbon of the long alkyl chains indexed with the letter *h* appears at 68.2 ppm. Besides, the signals at 62.5 and 43.1 ppm correspond to the *N*-(hydroxyethyl) moiety, the carbons of which were assigned as follows: the signal at 62.5 ppm is assigned to the methylene contiguous to the hydroxylic group (–OH), while the shielded signal at 43.1 ppm is attributed to the methylene contiguous to the amide group. Both signals were confirmed by HSQC NMR spectra (Fig. S3 in Supplementary information) and agreed with the characterization of similar 2-oxazoline derived dendrons previously described by Percec et al.^26,45^. The chemical shift (δ) of the benzylic methylenes depends on their relative position in the aromatic ring: Those in positions 3 and 5 appear at 71.4 ppm and corresponds to the lateral units (assigned with the letter *i’*), while the signal for the central unit appears downfield at 74.9 ppm (assigned with the letter *i*).

Furthermore, TAPAm structure was also characterized by means of FT-IR spectroscopy. Thus, the FT-IR spectrum of TAPAm shows a broad band at 3295 cm^−1^, which is attributed to the O–H stretching. Furthermore, a strong and thin band at 1614 cm^−1^ is attributed to the stretching of the carbonyl group of the amide. There are two more bands in the fingerprint region of the FT-IR that are associated to the O–H bond: the band at 1333 cm^−1^, which corresponds to the in-plane bending of the O–H bond, while the band at 1121 cm^−1^ can be assigned to the –(**C–O)**– stretching of the amide. The presence of all these bands corroborates that the amidation of TAPAm was successfully accomplished.

Due to the versatility of the oxazoline functional group, considerable efforts have been made to develop a broad scope of synthetic routes starting from esters^55,56^, nitriles^57^, carboxylic acids^58,59^ and *N*-(hydroxyethyl)amides^3,60,61^. From all of them, one of the most simple and well-known process is to convert the hydroxyl group of the *N*-(2-hydroxyethyl)amides into a good leaving group using thionyl chloride (SOCl_2_), followed by a basic treatment with NaHCO_3_ to isolate the 2-oxazoline derived ring^62^. Unfortunately, from what was observed when this reaction was carried out with TAPAm, the generated HCl in the reaction medium induced the cleavage of the ether benzylic moieties present in TAPAm. To suppress this side reaction, an excess of a non-nucleophilic base (DBU) was added before the addition of thionyl chloride to act as scavenger of the in-situ generated hydrochloric acid during the reaction as Šakalytė and co-workers did^31^. Nonetheless, the formation of TAPOx was not detected in the crude mixture while TAPAm was being consumed.

**Figure 5:**
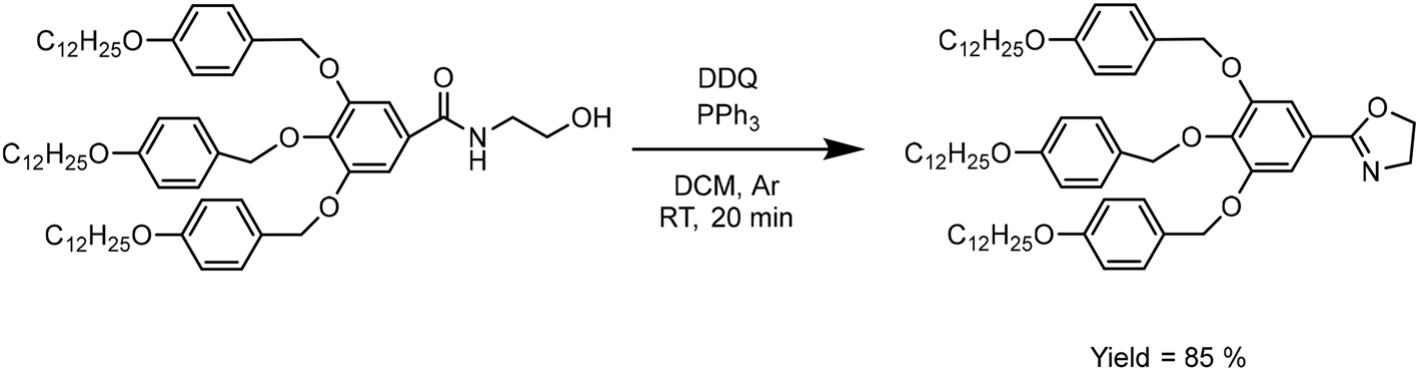
Synthesis of 2-(3,4,5-tris(4-dodecyloxybenzyloxy)phenyl)-2-oxazoline (TAPOx).

Thus, alternative reagents that favour the dehydrative cyclisation of amides should be considered. We selected the methodology reported by Xu and co-workers, who described a facile and rapid procedure for the synthesis of 2-oxazolines using PPh_3_-DDQ as oxidizing agent^63^. Among the advantages of this reaction, the authors highlighted its versatility (because it works well with distinct solvents such as DCM, 1,4-dioxane, THF, toluene), the mild reaction conditions (it takes place at room temperature) combined with the high yields of 2-oxazoline. Therefore, the synthesis of TAPOx was attempted using the PPh_3_-DDQ system (Fig. 5) and DCM as solvent. As Xu and co-workers previously demonstrated with various esters, we detected a complete conversion of the amide (TAPAm) by TLC after 20 min and TAPOx was isolated from the crude mixture by flash column chromatography with a high yield (85%).

**Figure 6:**
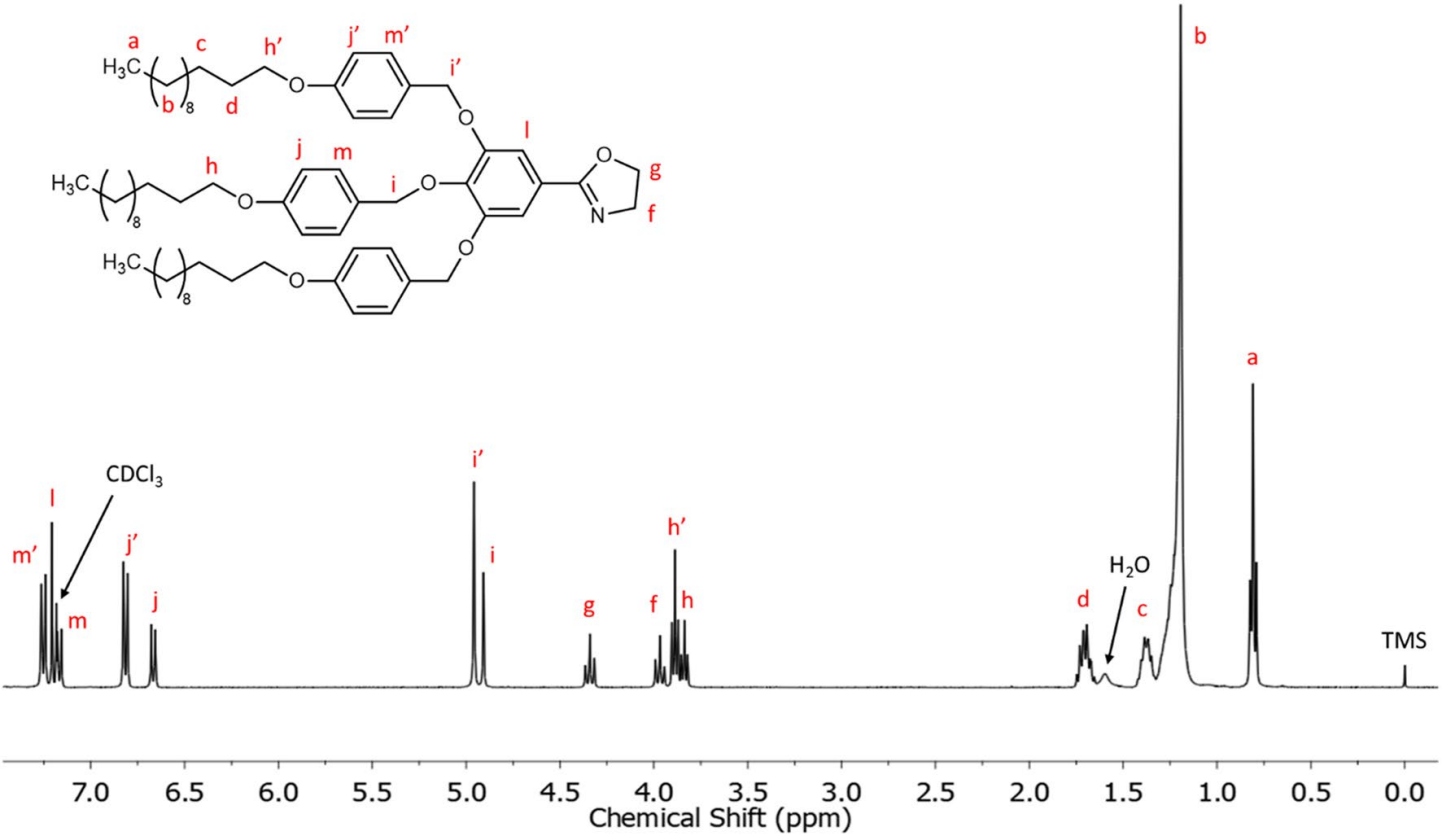
^1^H NMR spectrum in CDCl_3_ of TAPOx.

**Figure 7:**
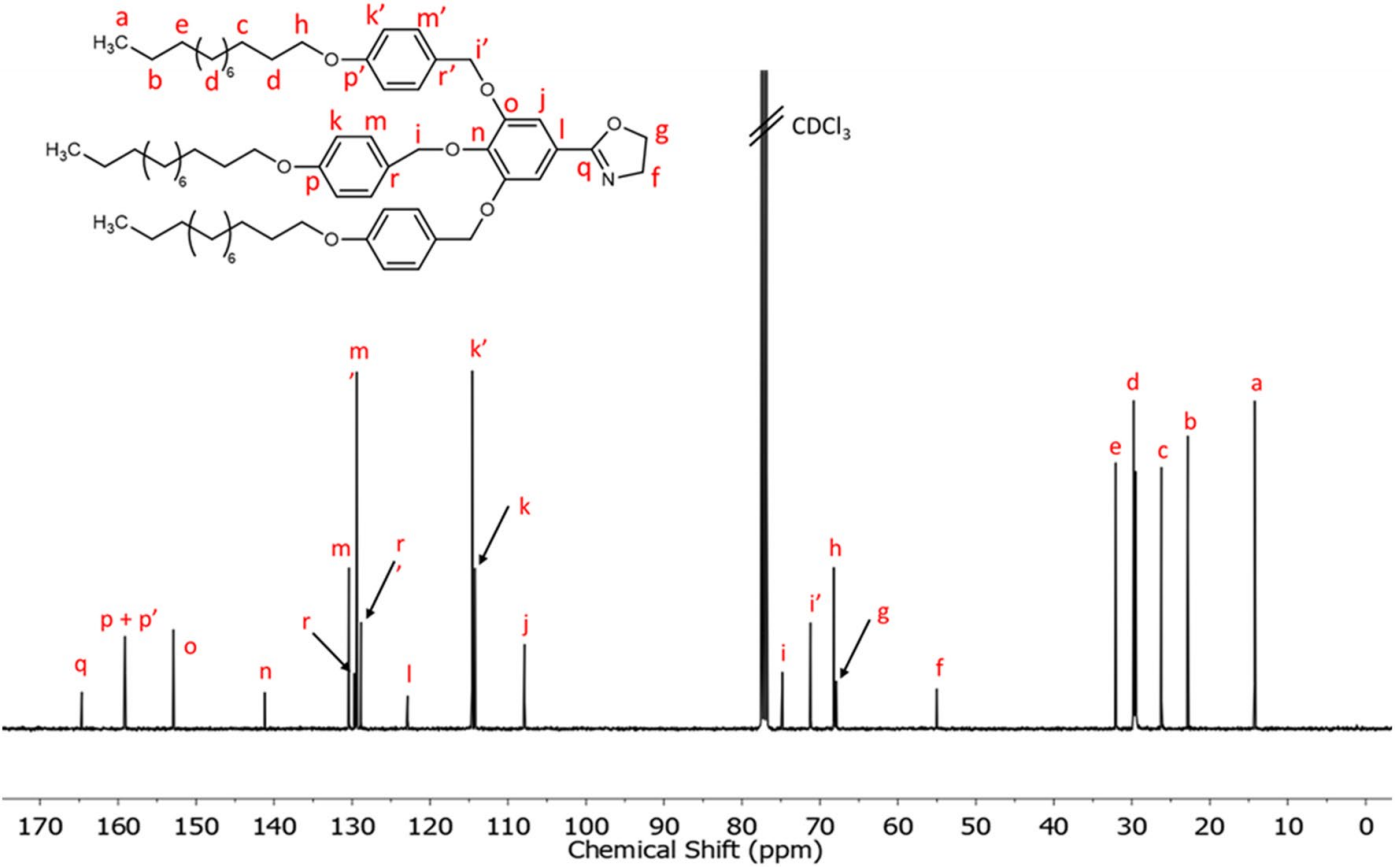
^13^C NMR spectrum in CDCl_3_ of TAPOx.

To validate the chemical structure of TAPOx, the obtained product was characterized by NMR and FT-IR spectroscopy. Figures 6 and 7 show the ^1^H NMR and ^13^C NMR spectra of TAPOx respectively, which were recorded using deuterated chloroform as solvent.

Starting with the downfield region in ^1^H NMR spectrum, the peaks between 7.25 and 6.67 ppm are assigned to the aromatic protons of the molecule. The two singlets that appear between 4.96 and 4.91 ppm are assigned to the benzylic methylenes of the dodecyloxybenzyloxy substituents. Furthermore, the two new triplets at 4.34 and 3.97 ppm are assigned to the methylenes of the formed oxazoline cycle: the triplet at 4.34 ppm is attributed to the methylene next to the oxygen, while the triplet centred at 3.97 ppm corresponds to the methylene contiguous to the nitrogen atom of the oxazoline ring as confirmed by COSY, HSQC and HMBC NMR spectra (Figs. S4, S5 and S6 in Supplementary information). Moreover, these assignments are in agreement with the characterization of 2-oxazoline monomers previously described by Percec and co-workers^26,45^. Furthermore, the two triplets centred at 3.89 and 3.84 ppm are attributed to methylene attached to the oxygen in long alkyl chains in lateral and central benzylic units, respectively. In the same manner than in the precursor TAPAm, signals between 1.70 and 0.81 ppm are attributed to the alkyl long chains of the dendron.

Related to ^13^C NMR, the carbon signal attributed to the –C=N– of the oxazoline ring appears at 164.7 ppm. The signals between 159.1 and 107.9 ppm are attributed to the aromatic carbons of the dendronized 2-oxazoline monomer. Is remarkable that the carbon of the aromatic ring linked to the oxazoline cycle appears slightly upfield in the spectrum compared to TAPAm precursor (122.9 ppm in front of the 129.5 ppm value observed in TAPAm). Same as TAPAm, the signals of the carbons that constitute the long alkyl chains appear at the expected region between 32.1 and 14.3 ppm (carbons labelled as a–e). The signals at 67.9 and 55.0 ppm are assigned to both methylene units of the 2-oxazoline ring, being the signal that appears at 67.9 ppm the one that corresponds to the methylene next to the oxygen, while the signal at 55.0 ppm is attributed to the methylene unit bonded to the nitrogen of the cycle. HSQC and HMBC NMR spectra allowed us to confirm the assignment of the characteristic signals of the 2-oxazoline ring (Figs. S5 and S6 in Supplementary information). Besides, the assignment agrees with the elucidation described in previous studies by Percec and co-workers^26,45^.

Moreover, the characteristic functional groups found in the chemical structure of TAPOx were characterized by FT-IR spectroscopy. In its FT-IR spectrum, no bands were observed above 3000 cm^−1^ and a new band attributed to the C=N stretching appears at 1643 cm^−1^, which is characteristic of the 2-oxazoline ring and confirms that the dehydrative cyclisation of *N*-(2-hydroxyethyl)-3,4,5–tris(4-dodecyloxybenzyloxy)benzamide was achieved^64^.

Finally, the direct synthesis of TAPOx from methyl 3,4,5-tris[*p*-(n-dodecan-1-yloxy)benzyloxy]benzoate (TAPEs) using lanthanide salts as catalyst was explored. The main advantage of this synthetic route is that only involves a single-step reaction as demonstrated by Zhou et al.^55^. In their work, the authors developed a simple and efficient method for the amidation of different carboxylic esters taking advantage of distinct inorganic catalyst (LaCl_3_, LaTf_3_ and SmCl_3_) obtaining 2-oxazolines in good yield when the reaction was performed under reflux of toluene. According to that, we carried out the one-pot synthesis of TAPOx from the starting ester dendron (TAPEs) using the conditions reported by Zhou and co-workers. In our case, different catalytic amounts of LaCl_3_ (5, 10, 20% mol respect to TAPEs) and LaTf_3_ (10% mol respect to TAPEs) were tested as catalyst. Nevertheless, the formation of 2-oxazoline compound was never detected in the reaction medium even though the formation of the amide (TAPAm) was observed when lanthanum triflate was employed. These observations agree with the results presented by Morimoto and co-workers, in which they reported the amidation of esters using 5% mol of LaTf_3_ in mild conditions with good yields (≥ 80%)^65^. However, we could not isolate the TAPAm fraction from the crude mixture even by flash column chromatography due to its complexity and the low amount of TAPAm that it presented.

To summarize the reactions that were carried out in the synthesis of TAPOx, Table 2 presents the starting materials, the solvent and the reaction conditions employed in all the reactions performed. Furthermore, the obtained yield and general remarks have been also shown.

**Table 2:** Starting materials, solvent, reaction conditions and results obtained in the chemical reactions performed in the synthesis of TAPOx.

Starting materials (mmols)	Solvent	Temperature (°C)	Time (h)	Yield (%)/comments
TAPAm (0.38); SOCl_2_ (1.15); DBU (2.31)	DCM	RT^a^	2	No TAPOx was detected in the crude mixture after the complete consumption of TAPAm
TAPAm (0.66); PPh_3_ (0.98); DDQ (0.98)	Anhydrous DCM	RT^a^	0.33	85/TAPOx was obtained with high yield. Nevertheless, a flash column chromatography is required to obtain pure TAPOx
TAPEs (0.99); ethanolamine (2.48); n-BuLi (2.25); anhydrous LaCl_3_ (0.05)	Anhydrous toluene	100	27	No TAPOx was detected in the crude mixture after complete consumption of TAPEs despite the formation of TAPAm was detected
TAPEs (0.99); ethanolamine (2.48); n-BuLi (2.25); anhydrous LaCl_3_ (0.10)	Anhydrous toluene	100	24	No TAPOx was detected in the crude mixture after complete consumption of TAPEs despite the formation of TAPAm was detected
TAPEs (1.00); ethanolamine (2.50); n-BuLi (2.25); anhydrous LaCl_3_ (0.20)	Anhydrous toluene	100	22	No TAPOx was detected in the crude mixture after complete consumption of TAPEs despite the formation of TAPAm was detected
TAPEs (0.99); ethanolamine (2.48); n-BuLi (2.25); anhydrous LaTf_3_ (0.10)	Anhydrous toluene	100	22	No TAPOx was detected in the crude mixture after complete consumption of TAPEs despite the formation of TAPAm was detected

^a^RT, room temperature = 20 ± 3 °C.

**Figure 8:**
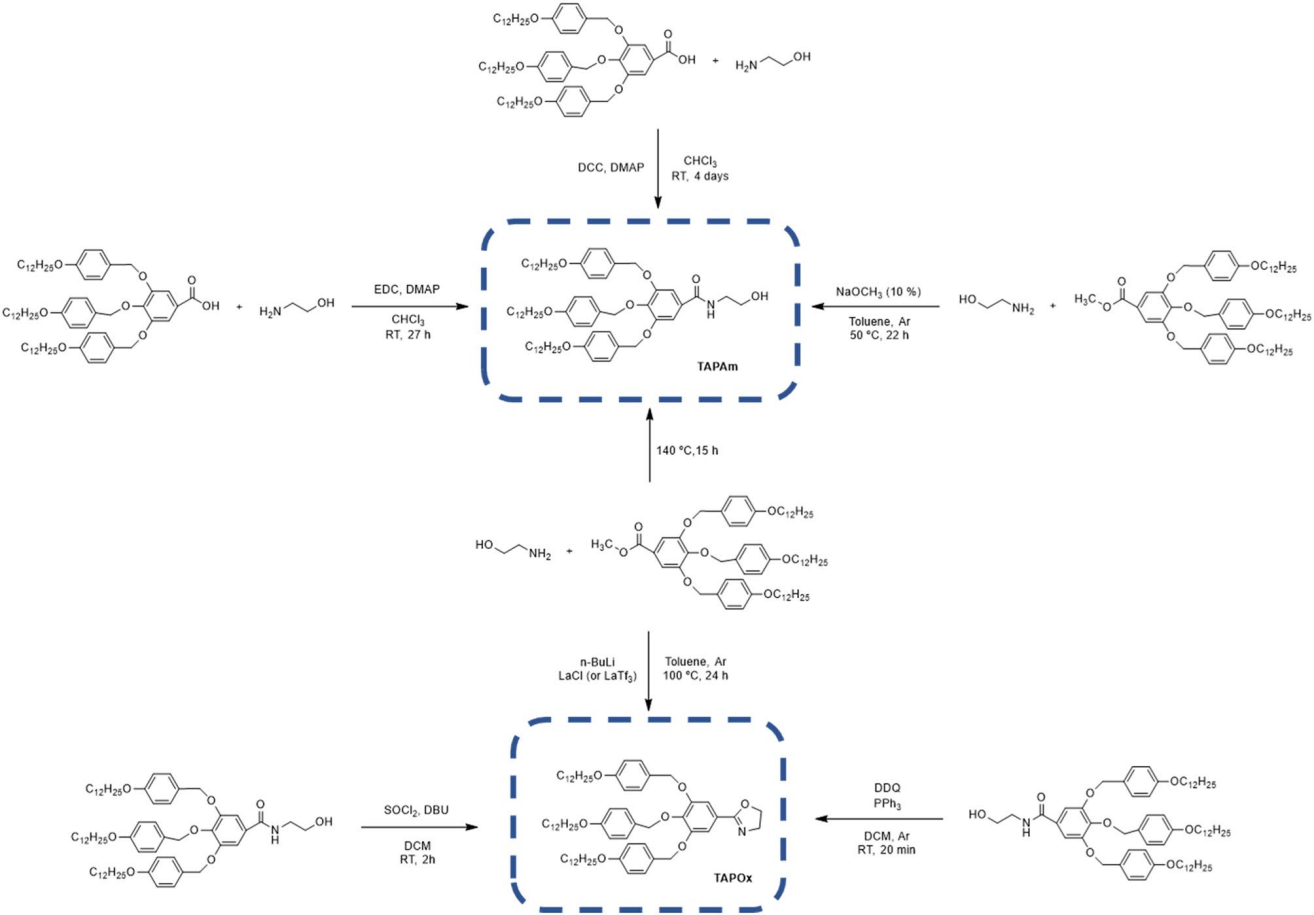
General scheme of the synthetic routes addressed in the synthesis of dendronized TAPOx monomer.

At the end, Fig. 8 outlined all the synthetic routes that we had explored to obtain TAPOx monomer.

### Thermal and mesomorphic characterization of TAPAm and TAPOx

**Figure 9:**
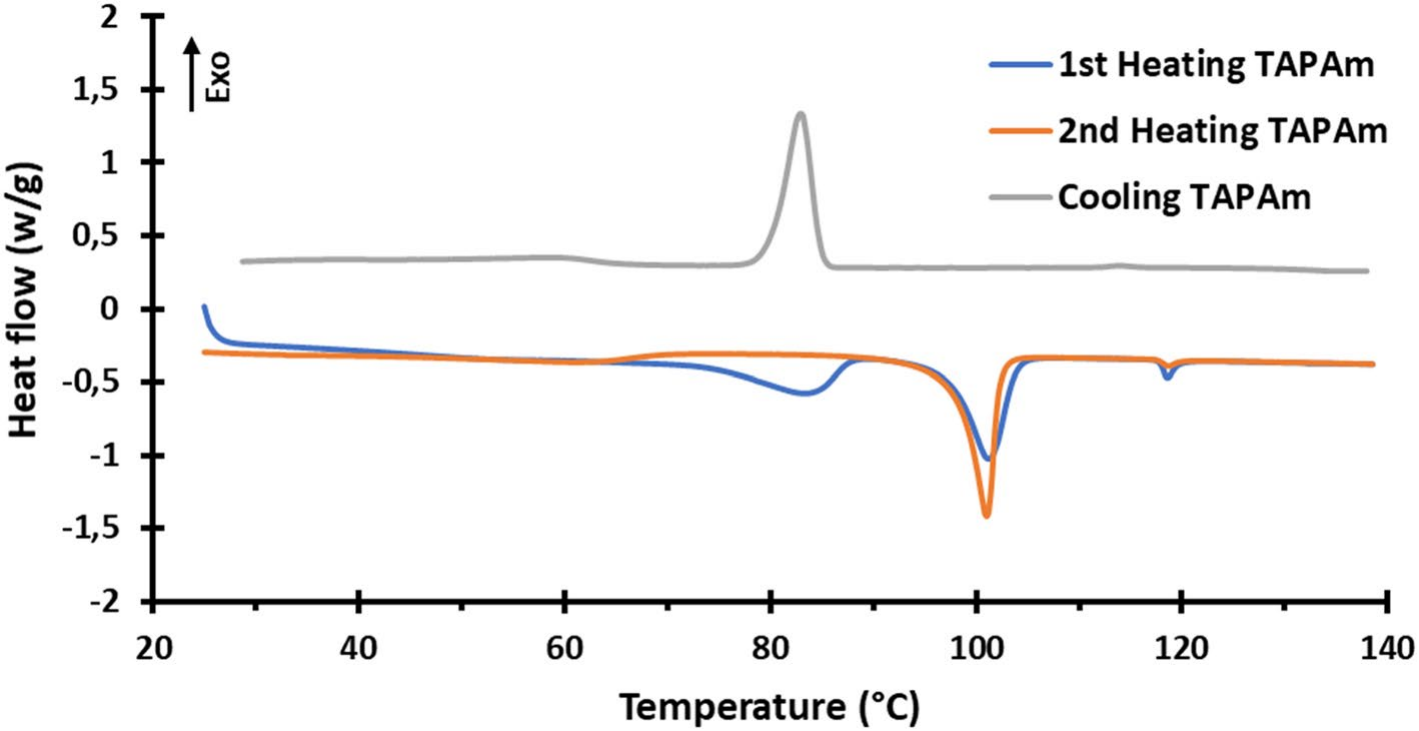
Calorimetric analysis of TAPAm; (blue) first heating, (orange) second heating, and (grey) cooling. Scan rate: 10 °C/min.

Thermal and mesomorphic characterization of TAPAm and TAPOx was performed by DSC, TGA, POM and XRD experiments. DSC study of TAPAm is depicted in Fig. 9. First and second heating scans are shown as well as the first cooling scan. The first heating scan shows a broad endotherm at 84 °C, followed by a second sharper endotherm at 101 °C; finally, a small endotherm is shown at 118 °C. On cooling scan, we only observe an exotherm centred at about 83 °C. At second heating scan, we are able only to see two endotherms, the first one centred at 101 °C and the second one at 118 °C. As we could observe, after the clearing of the substrate the first endotherm was no longer observed, showing only these two peaks which are compatible with the occurrence of a mesophase in the range of temperatures from 101 to 118 °C. Table 3 shows the associated enthalpies for each phase transition and clearing temperature for TAPAm on first and second heating scans.

**Table 3:** Enthalpies of phase transitions detected in the case of TAPAm.

	1st heating		2nd heating	
	T (°C)	ΔH (kJ/mol)	T (°C)	ΔH (kJ/mol)
1st Endotherm	84	11.0	–	–
2nd Endotherm	101	19.0	101	21.8
T_c_	118	0.8	118	0.3

**Figure 10:**
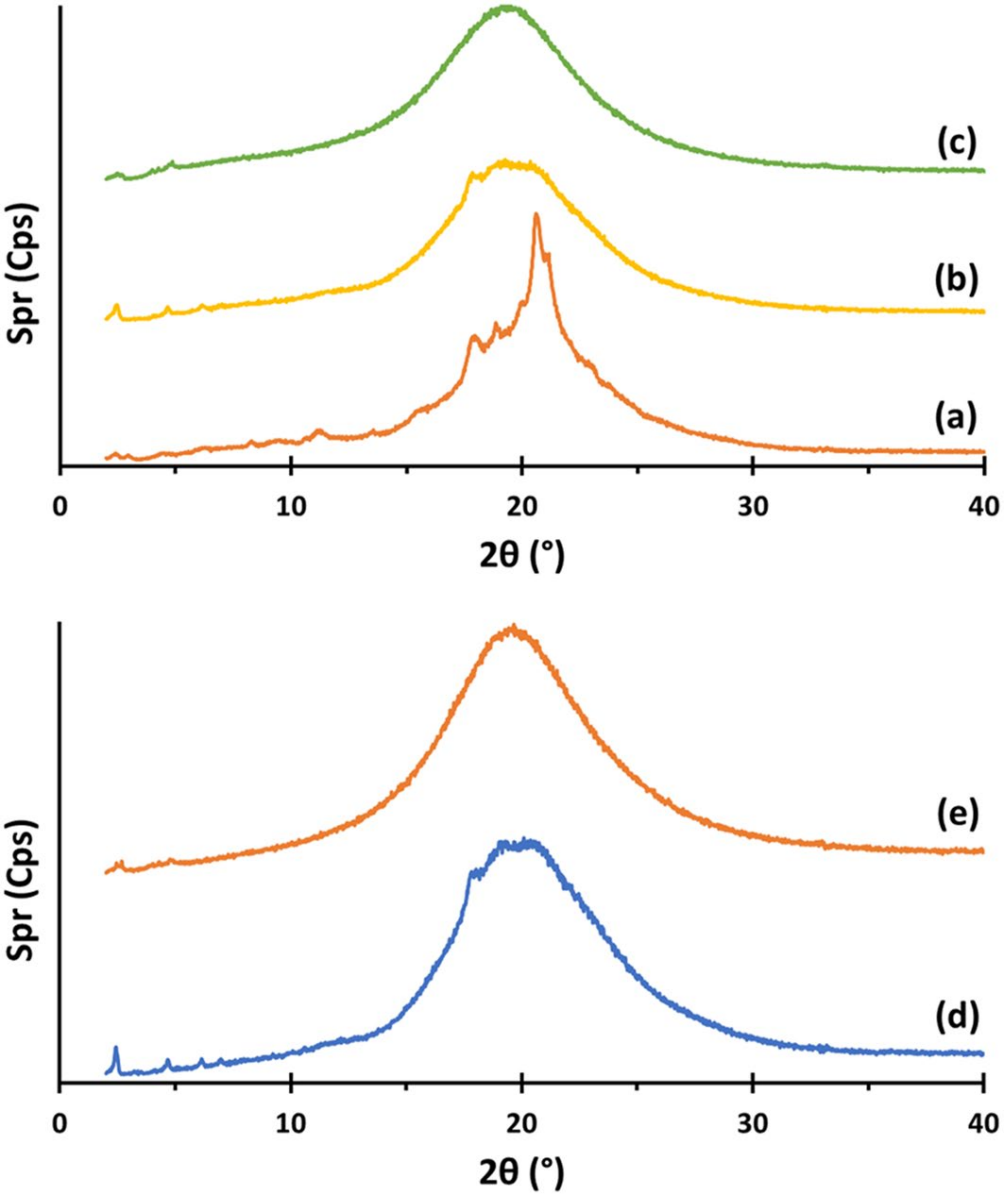
XRD patterns of TAPAm recorded on heating at: (**a**) 70 °C, (**b**) 90 °C and (**c**) 110 °C on first heating; and (**d**) 80 °C and (**e**) 110 °C on second heating.

**Figure 11:**
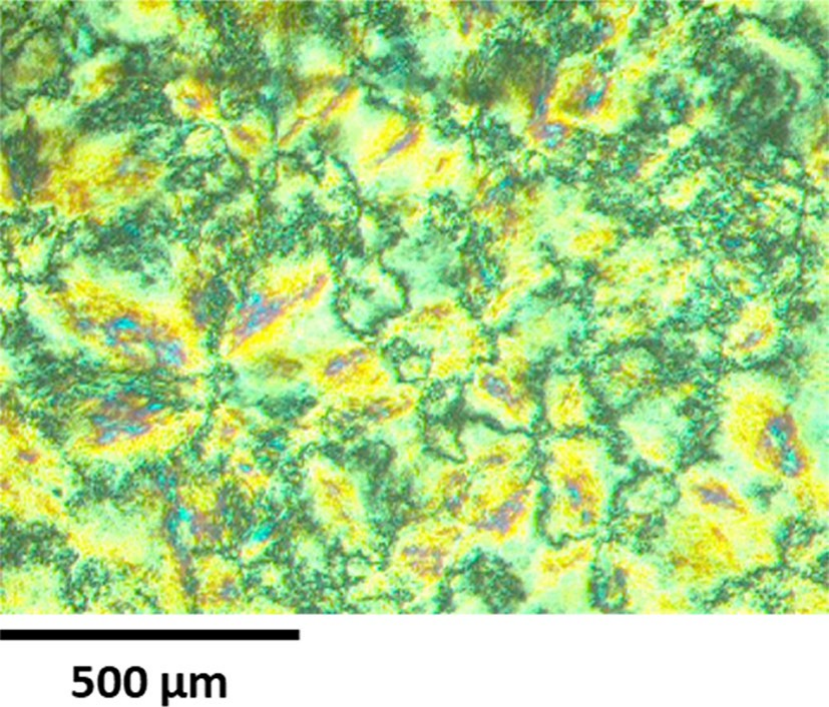
Optical micrographs between crossed polars of TAPAm recorded during the first heating at 90 °C.

Based on DSC and XRD experiments (Fig. 10), we observed a metastable crystalline phase below first endotherm on first heating, since this phase was not observed at the second heating. On the other hand, we envisioned a highly ordered columnar phase between 84 and 101 °C at first heating. POM observation, in this range of temperatures, put into evidence a pseudo-focal conic fan-shaped texture (Fig. 11). XRD pattern recorded at 90 °C (Fig. 10b) shows three main peaks centred at 2θ = 2.4° (d = 36 Å), 4.6° (d = 19 Å), 6.2° (d = 14 Å) and an unsymmetrical broad halo at 2θ = 20° (d = 4 Å approximately) showing shoulders. The lower spacing observed in the XRD pattern should correspond to the planar distance between dendrons and the higher spacing might correspond to the lateral distance between columns. It is noteworthy that for the second heating only this phase was observed below the endotherm centred at 101 °C.

For the range of temperature between 101 and 118 °C, we observed the presence of a mesophase. This was confirmed by XRD experiments, with a pattern recorded at 110 °C (Fig. 10c,e); we saw four main peaks centered at 2θ = 2.6° (d = 34 Å), 3.9° (d = 22 Å), 4.3° (d = 20 Å), 4.9° (d = 18 Å) and a symmetrical broad halo at 2θ = 20° (d = 4 Å approximately). Same as before, the lower spacing can be attributed to the distance between dendrons and the higher one to the lateral distance between columns. In this case, the phase observed is quite disordered, as we could inferred by DSC, where the enthalpy of the endotherm was only 0.8 kJ/mol (Table 3). Furthermore, on second heating we observed the same behaviour as first heating in the range of temperatures from 101 to 118 °C. It is remarkable that on cooling from the melt state we did not observe the formation of this phase by DSC nor POM. Therefore, it is suggested that TAPAm should present a monotropic columnar mesophase in the range of temperature from 101 to 118 °C.

**Figure 12:**
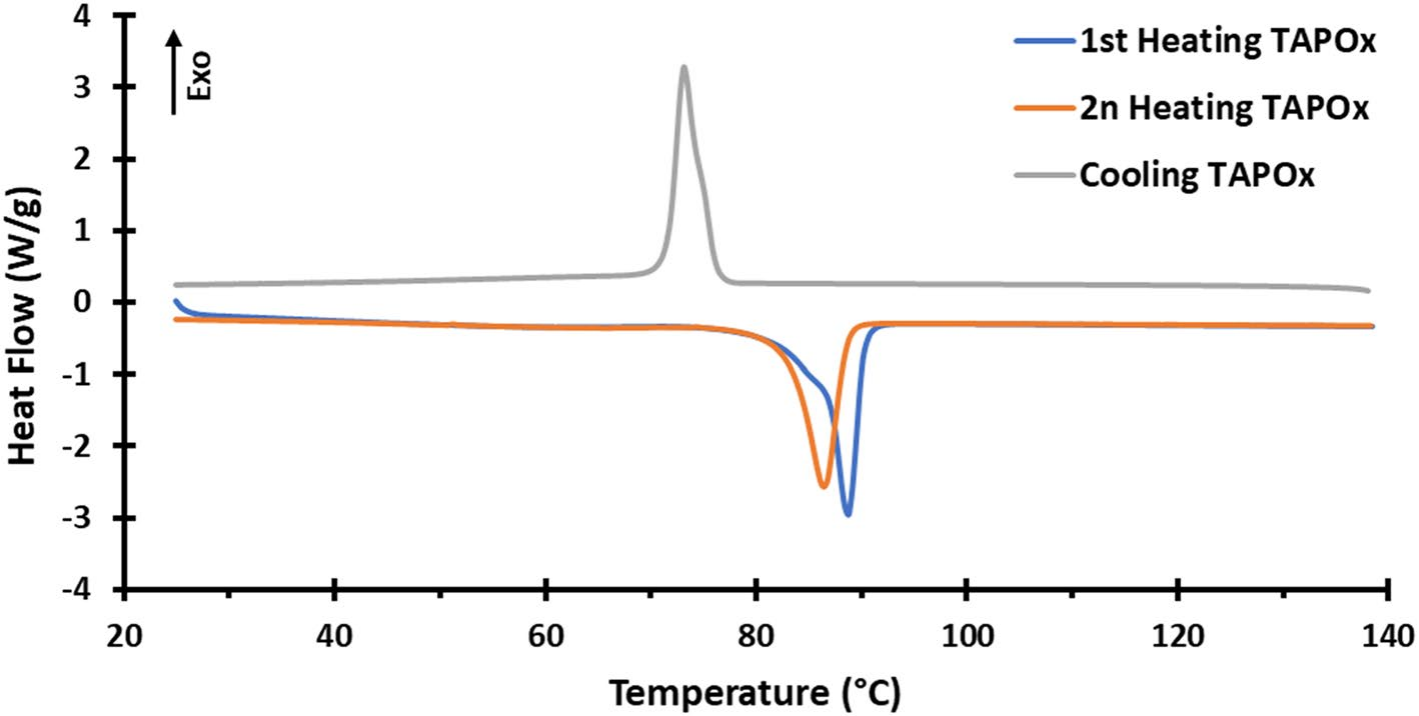
Calorimetric analysis of TAPOx; (blue) first heating, (orange) second heating, and (grey) cooling. Scan rate: 10 °C/min.

Calorimetric study by DSC of TAPOx is depicted in Fig. 12, showing first and second heating and cooling scans. On heating we observe one endotherm centered at 89 °C for the first heating and 86 °C for the second heating, which can be attributed to the melting of TAPOx, meanwhile on cooling we observe a peak centered at 73 °C, which can be assigned to the crystallization of TAPOx. According to POM experiments, on heating we did not observe a mesophase below melting point and an isotropic liquid was observed above the endotherm. Moreover, on cooling we did not notice the presence of a mesophase from the melt state. This behaviour was also confirmed by XRD experiments, showing a crystalline phase below the melting point and an isotropic liquid above the endotherm (Fig. S7). Therefore, TAPOx shows no mesomorphic behaviour. Thus, it seems that the stiffness of the oxazoline ring strongly difficults the packing of tapered groups, avoiding the formation of columnar phases.

**Figure 13:**
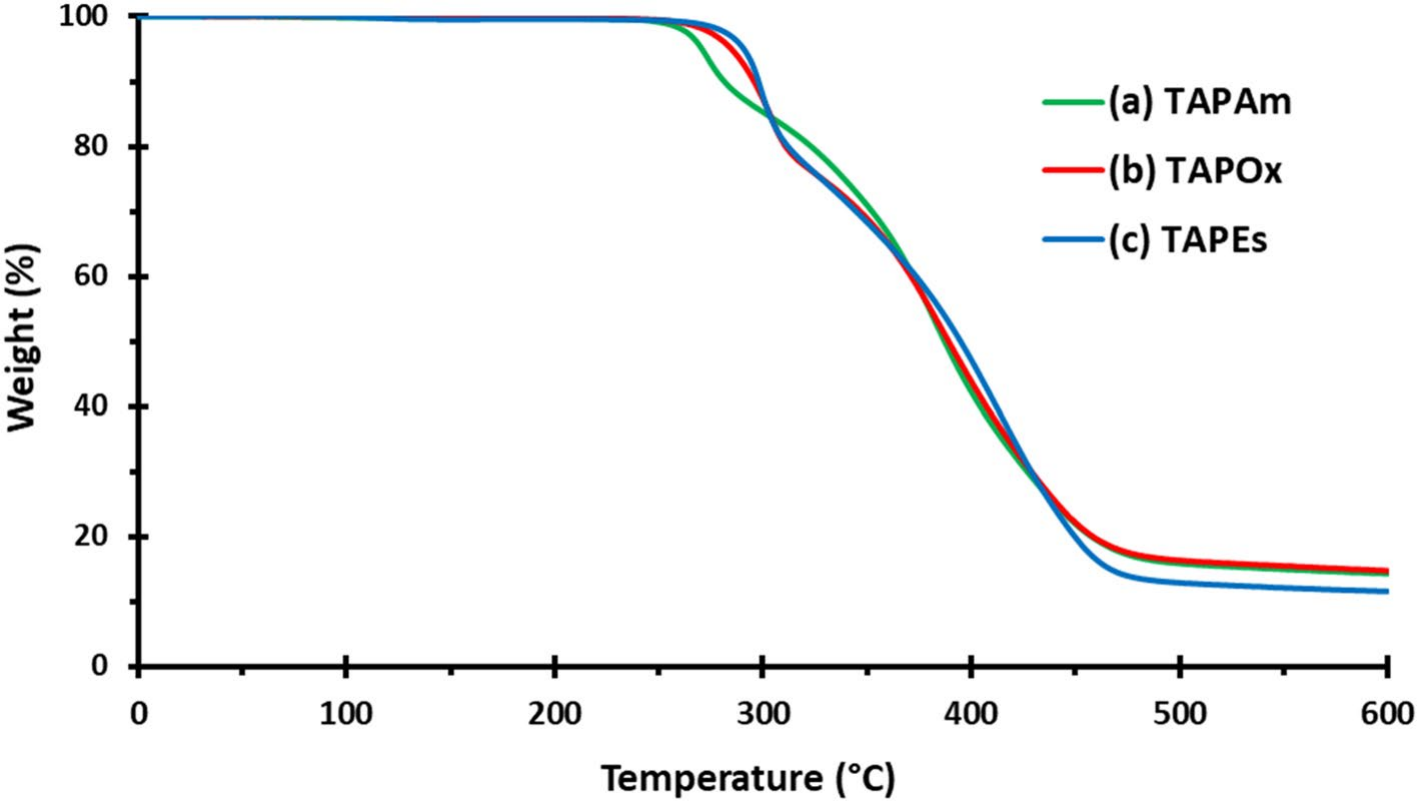
TGA thermogram of (**a**) TAPAm, (**b**) TAPOx and (**c**) TAPEs recorded at a heating rate of 10 °C/min in nitrogen atmosphere.

Thermal stability of TAPAm and TAPOx was studied by TGA (Fig. 13). Onset of thermal weight loss (determined as the temperature corresponding to 5% mass loss) was 271 °C for TAPAm with 14% of remaining char yield at 600 °C. TAPOx is slightly more stable, with an onset of the thermal weight loss of 285 °C and a similar char yield at 600 °C (15%) compared with its amide precursor. In the case of TAPEs, it presents the higher onset of thermal weight loss of the 3 dendronized compounds: 291 °C, but the smallest remaining char yield (12%). Besides, the thermal degradation of these three dendronized compounds showed a two-step weight loss, as confirmed by DGTA curves (Fig. S8). The presence of a hydroxyl group in TAPAm chemical structure can lead to an easy dehydration that could induce the thermal degradation of TAPAm at a lower temperature than TAPOx and TAPEs, which DTGA curve showed the same trend.

### Preliminary polymerization study

As aforementioned, it is described that 2-substituted-2-oxazolines polymerize by living cationic ring opening polymerization (CROP). Many procedures to polymerize these compounds can be founded in literature, i.e. Percec and co-workers described the polymerization of 2-[3,4-bis(*n*-alkan-1-yloxy)phenyl]-2-oxazolines using *o-*dichlorobenzene as a solvent at 110 °C and bulk polymerization at 160 °C, with methyl triflate (MeOTf) as an initiator^26^. Apart from MeOTf, a wide variety of initiators have been used, such as sulfonate esters, Lewis acids and alkyl halides among others^10^. Furthermore, several terminating agents have been employed in this type of polymerizations, such as primary, secondary and aromatic amines, hydroxide salts and alcohols, among others^3^**.**

Herein, we describe a preliminary study of polymerization of TAPOx. All the conditions tested in each polymerization of TAPOx are summarized in Table 4. On our first attempt, we try to synthetize our polymer using methyl tosylate (MeOTs) as an initiator and toluene as a solvent under reflux conditions. We monitored the reaction by ^1^H NMR; nevertheless, after 12 days of reaction the product was not noticed, and only unreacted monomer was observed at the reaction medium. On a second attempt, we follow the procedure described by Pásztói et al.^66^, where benzotrifluoride was used as an environmentally less harmful solvent to polymerize 2-ethyl-2-oxazoline at 100 °C using MeOTs as an initiator. Unfortunately, following the same trend as the first attempt, the polymer was not detected after 10 days of reaction and only unreacted TAPOx was present at the reaction medium.

**Table 4:** Polymerization reaction conditions of TAPOx monomer (initiator, terminating agent, solvent, and reaction conditions) and the obtained results.

Entry	Initiator (% mol)/terminating agent	Solvent	Reaction conditions	Comments/yield (%)
1	MeOTs (1)/–	Anhydrous toluene	130 °C; 12 days	Virgin monomer was recovered
2	MeOTs (1)/–	Anhydrous benzotrifluoride	90 °C; 10 days	Virgin monomer was recovered
3	MeOTs (1)/Dodecylamine	–	130 °C; 2 h	62

**Figure 14:**
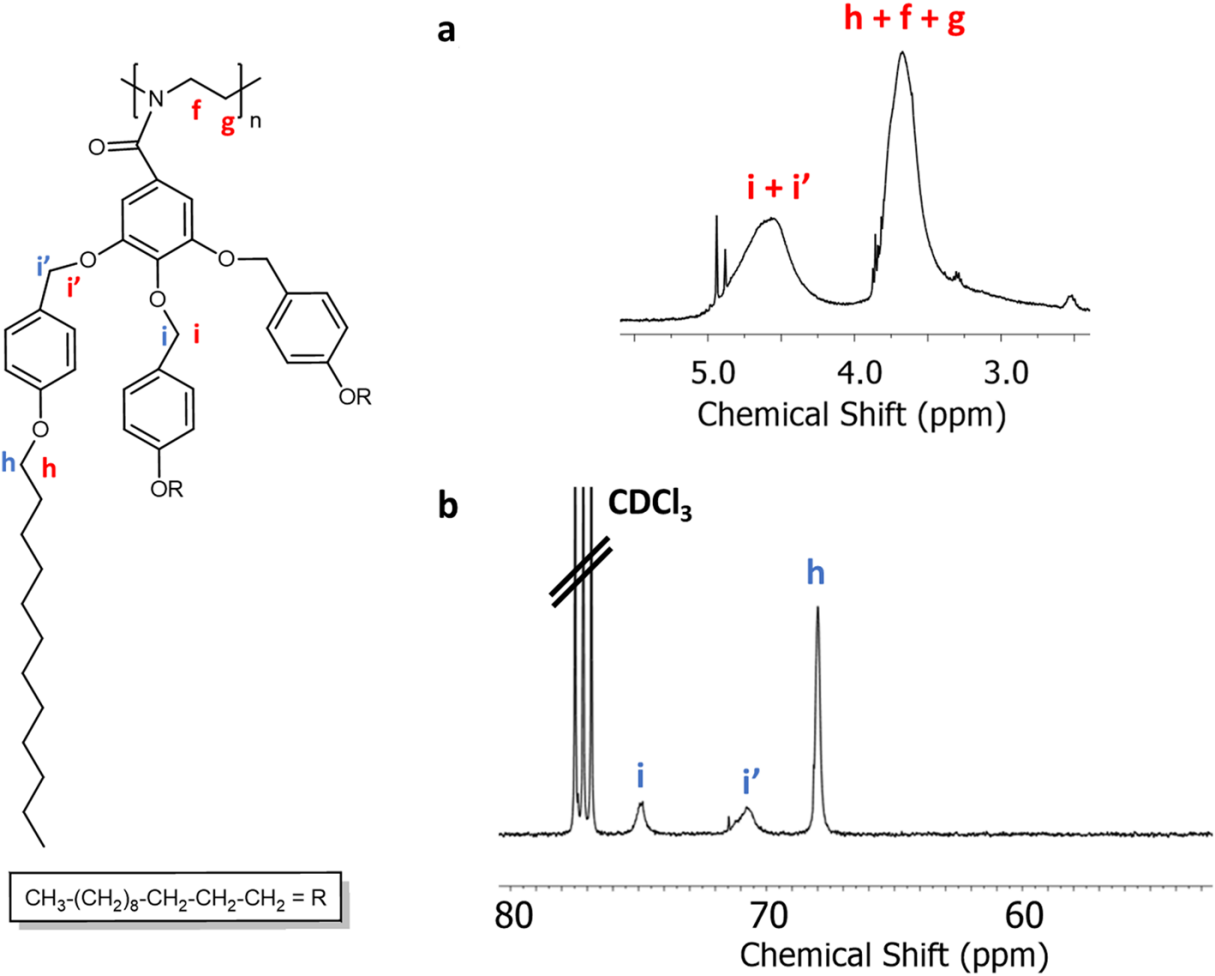
Zooms of the NMR spectra of the polyoxazoline derived from TAPOx: (**a**) ^1^H NMR spectrum between 2.5 and 5.5 ppm (signals labelled in red); and (**b**) ^13^C NMR spectrum between 50 and 80 ppm (signals labelled in blue).

Therefore, we decided to carry out a bulk polymerization of TAPOx at 130 °C using MeOTs as an initiator. In this case, we could confirm the total conversion of the monomer through ^1^H NMR analysis (Fig. 14a) and ^13^C NMR (Fig. 14b) experiments. Regarding ^1^H NMR analysis, the characteristic signals corresponding to both methylenes in the oxazoline ring at 4.34 and 3.97 ppm (see Fig. 6) disappear and a broad signal corresponding to the main chain of polyoxazoline appears centred at 3.67 ppm. Furthermore, according to ^13^C NMR experiments, full conversion of TAPOx was also confirmed, since the signal corresponding to the methylene contiguous to nitrogen inside the oxazoline ring, at 55.0 ppm (see Fig. 7), also disappeared. Despite the full conversion of the monomer was confirmed by NMR, the yield of the polymerization after work-up was only 62%. This fact indicates us that we must optimize the purification process. Besides, based on the relationship between the signals of the terminating agent added (dodecylamine) and the polymer in ^1^H NMR, we found that only oligomers were synthetized; in fact, pentamers were obtained.

As a complementary analysis, we decided to observe the synthesized oligomers by polarized optical microscopy in order to see whether it presents a mesophase. We found that, on heating, a mesophase was noticed, actually, an homeotropic texture was observed.

It is clear that, the polymerization of TAPOx deserves a deeper study, in order to find the conditions to get a columnar polyoxazolines of quite high molecular weight. This study is in progress and will be the subject of a forthcoming paper.

## Conclusions

A novel mesogenic oxazoline, 2-(3,4,5-tris(4-dodecyloxybenzyloxy)phenyl)-2-oxazoline (TAPOx), was successfully synthetized in 2 steps. Firstly, the amidation in mild conditions of methyl 3,4,5-tris[*p*-(n-dodecan-1-yloxy)benzyloxy]benzoate (TAPEs) using 10% mol of sodium methoxide as catalyst, since a high yield of the resulting amide (TAPAm) is obtained due to its easy purification by recrystallization (92%). The second step involves a dehydrative cyclisation of the amide using PPh_3_-DDQ system as oxidizing agent. Although the corresponding 2-oxazoline derived monomer was purified by flash column chromatography, TAPOx was also obtained with a good yield (85%).

Thermal and mesomorphic studies showed that a monotropic columnar mesophase was observed on heating for TAPAm in the range of temperatures from 101 to 118 °C, meanwhile any liquid crystalline texture was detected in the case of TAPOx.

Furthermore, a preliminary research of the cationic ring opening polymerization of TAPOx was carried out, from which it was deduced that a profound investigation should be addressed to find the optimal conditions to get columnar polyoxazolines with high molecular weight.

### Supplementary Information

Supplementary Information.

## References

[1] Andreasch R (1884). Zur Kenntniss des Allylharnstoffs. Monats. Chem..

[2] Gabriel S (1889). Zur Kenntniss des Bromäthylamins. Ber. Dtsch. Chem. Ges..

[3] Glassner M, Vergaelen M, Hoogenboom R (2018). Poly(2-oxazoline)s: A comprehensive overview of polymer structures and their physical properties. Polym. Int..

[4] Varanaraja Z, Kim J, Becer CR (2021). Poly(2-oxazine)s: A comprehensive overview of the polymer structures, physical properties and applications. Eur. Polym. J..

[5] Nishiyama H (1989). Chiral and C2-symmetrical Bis( oxazolinylpyridine )rhodium( III) complexes: Effective catalysts for asymmetric hydrosilylation of ketones. Organometallics.

[6] Do HQ, Chandrashekar ERR, Fu GC (2013). Nickel/bis(oxazoline)-catalyzed asymmetric negishi arylations of racemic secondary benzylic electrophiles to generate enantioenriched 1,1- diarylalkanes. J. Am. Chem. Soc..

[7] Saravanan P, Corey EJ (2003). A short, stereocontrolled, and practical synthesis of α- methylomuralide, a potent inhibitor of proteasome function. J. Org. Chem..

[8] Meyers AI, Temple DL, Nolen RL, Mihelich ED (1974). Oxazolines: IX Synthesis of homologated acetic acids and esters. J. Org. Chem..

[9] Kobayashi S (1990). Ethylenimine polymers. Prog. Polym. Sci..

[10] Aoi K, Okada M (1996). Polymerization of oxazolines. Prog. Polym. Sci..

[11] Verbraeken B, Monnery BD, Lava K, Hoogenboom R (2017). The chemistry of poly(2-oxazoline)s. Eur. Polym. J..

[12] Moreadith RW (2017). Clinical development of a poly(2-oxazoline) (POZ) polymer therapeutic for the treatment of Parkinson’s disease – Proof of concept of POZ as a versatile polymer platform for drug development in multiple therapeutic indications. Eur. Polym. J..

[13] Li, X. & Yu, H. Side Chain Liquid Crystalline Polymers: Advances and Applications. in *Liquid Crystalline Polymers, Volume 2 - Processing and Applications* (Eds. Thakur, V. K.; Kessler, M. R.) Vol. 2, pp. 369–386 (Springer Science+Business Media, 2015).

[14] Vicari, L. *Optical applications of liquid crystals* (Taylor & Francis, 2010).

[15] Bustamante EAS, Haase W (1997). Synthesis and characterization of new liquid crystalline monomers for non-linear optics: X-ray study of re-entrant nematic behaviour with smectic-like fluctuations of C-type. Liq. Cryst..

[16] Shih HF, Wu DY, Tien CL, Dai CL (2013). Optical compensator with switchable modes using polymer stabilized liquid crystals. Opt. Rev..

[17] Gin DL, Noble RD (2011). Designing the next generation of chemical separation membranes. Science.

[18] Zhou M (2007). New type of membrane material for water desalination based on a cross-linked bicontinuous cubic lyotropic liquid crystal assembly. J. Am. Chem. Soc..

[19] Cho BK (2014). Nanostructured organic electrolytes. RSC Adv..

[20] Sakuda J (2015). Liquid-crystalline electrolytes for lithium-ion batteries: Ordered assemblies of a mesogen-containing carbonate and a lithium salt. Adv. Funct. Mater..

[21] Sergeyev S, Pisula W, Geerts YH (2007). Discotic liquid crystals: A new generation of organic semiconductors. Chem. Soc. Rev..

[22] Funahashi M (2009). Development of liquid-crystalline semiconductors with high carrier mobilities and their application to thin-film transistors. Polym. J..

[23] Rodríguez-Parada JM, Percec V (1987). Synthesis and characterization of liquid crystalline poly(N- acylethyleneimine)s. J. Polym. Sci. Part A Polym. Chem..

[24] Percec V, Johansson G, Schlueter D, Ronda JC, Ungar G (1996). Molecular recognition directed self-assembly of tubular supramolecular architectures from building blocks containing monodendrons as exo-receptors and crown- or pseudo-crown-ethers as endo-receptors. Macromol. Symp..

[25] Yeardley DJP, Ungar G, Percec V, Holerca MN, Johansson G (2000). Spherical supramolecular minidendrimers self-organized in an ’inverse micellar’-like thermotropic body-centered cubic liquid crystalline phase. J. Am. Chem. Soc..

[26] Percec V (2001). Poly(oxazolines)s with tapered minidendritic side groups: The simplest cylindrical models to investigate the formation of two-dimensional and three-dimensional order by direct visualization. Biomacromol.

[27] Percec V, Holerca MN, Uchida S, Yeardley DJP, Ungar G (2001). Poly(oxazoline)s with tapered minidendritic side groups as models for the design of synthetic macromolecules with tertiary structure: A demonstration of the limitations of living polymerization in the design of 3-D structures based on single polymer chain. Biomacromol.

[28] Kim KM, Imai Y, Naka K, Chujo Y (2000). Synthesis and characterization of new side-chain liquid crystalline polyoxazolines. Polym. J..

[29] Bhosale S, Rasool M, Reina J, Giamberini M (2013). New liquid crystalline columnar poly(epichlorohydrin-co-ethylene oxide) derivatives leading to biomimetic ion channels. Polym. Eng. Sci..

[30] Montané X, Bhosale SV, Reina JA, Giamberini M (2015). Columnar liquid crystalline polyglycidol derivatives: A novel alternative for proton-conducting membranes. Polymer.

[31] Šakalytė A, Reina JA, Giamberini M (2013). Liquid crystalline polyamines containing side dendrons: Toward the building of ion channels based on polyamines. Polymer.

[32] Montané X (2016). Advances in the design of self-supported ion-conducting membranes—New family of columnar liquid crystalline polyamines: Part 1: Copolymer synthesis and membrane preparation. Polymer.

[33] Montané X (2016). Advances in the design of self-supported ion-conducting membranes—New family of columnar liquid crystalline polyamines: Part 2: Ion transport characterisation and comparison to hybrid membranes. Polymer.

[34] Šakalytė A, Giamberini M, Reina JA (2014). Synthesis and characterisation of a monotropic dendritic liquid crystalline aziridine monomer. Liq. Cryst..

[35] Perrin, D. D. & Armarego, W. L. F. *Purification of Laboratory Chemicals*. (Pergamon Press, 2009).

[36] Ronda JC, Reina JA, Giamberini M (2003). Self-organized liquid-crystalline polyethers obtained by grafting tapered mesogenic groups onto poly(epichlorohydrin): Toward biomimetic ion channels 2. J. Polym. Sci. Part A Polym. Chem..

[37] Bernhard N, Wolfgang S (1978). Simple method for the esterification of carboxylic acids. Angew. Chemie Int. Edit..

[38] Holmberg K, Hansen B (1979). Ester synthesis with dicyclohexylcarbodiimide improved by acid catalysts. Acta Chem. Scand. B.

[39] El-Faham A, Albericio F (2011). Peptide coupling reagents, more than a letter soup. Chem. Rev..

[40] Carpino LA, El-Faham A (1999). The diisopropylcarbodiimide/1-hydroxy-7-azabenzotriazole system: Segment coupling and stepwise peptide assembly. Tetrahedron.

[41] Sheehan J, Cruickshank P, Boshart G (1961). Notes: A convenient synthesis of water-soluble carbodiimides. J. Org. Chem..

[42] Wigman L (2014). Byproducts of commonly used coupling reagents: Origin, toxicological evaluation and methods for determination. Am. Pharm. Rev..

[43] Klausner YS, Bodansky M (1972). Coupling reagents in peptide synthesis. Synth. Stuttgart.

[44] Smith, M. B. *Advanced Organic Chemistry: Reactions, mechanisms, and structure*. (John Wiley & Sons, Inc., 2020).

[45] Holerca MN (2018). Dendronized poly(2-oxazoline) displays within only five monomer repeat units liquid quasicrystal, A15 and σ Frank-Kasper phases. J. Am. Chem. Soc..

[46] Ishihara K, Kuroki Y, Hanaki N, Ohara S, Yamamoto H (1996). Antimony-templated macrolactamization of tetraamino esters. Facile synthesis of macrocyclic spermine alkaloids, (±)-buchnerine, (±)-verbacine, (±)-verbaskine, and (±)-verbascenine. J. Am. Chem. Soc..

[47] Han C, Lee JP, Lobkovsky E, Porco JA (2005). Catalytic ester-amide exchange using group (IV) metal alkoxide-activator complexes. J. Am. Chem. Soc..

[48] Movassaghi M, Schmidt MA (2005). N-heterocyclic carbene-catalyzed amidation of unactivated esters with amino alcohols. Org. Lett..

[49] Price KE (2009). Mild and efficient DBU-catalyzed amidation of cyanoacetates. Org. Lett..

[50] Sabot C, Kumar KA, Meunier S, Mioskowski C (2007). A convenient aminolysis of esters catalyzed by 1,5,7-triazabicyclo[4.4.0]dec-5-ene (TBD) under solvent-free conditions. Tetrahedron Lett..

[51] Yang X, Birman VB (2009). Acyl transfer catalysis with 1,2,4-Triazole anion. Org. Lett..

[52] Meher LC, Vidya Sagar D, Naik SN (2006). Technical aspects of biodiesel production by transesterification—A review. Renew. Sustain. Energy Rev..

[53] Ohshima T (2012). Sodium methoxide: A simple but highly efficient catalyst for the direct amidation of esters. Chem. Commun..

[54] Johansson G, Percec V, Ungar G, Abramic D (1994). Molecular recognition directed self-assembly of tubular liquid crystalline supramolecular architectures from taper shaped (15-Crown-5)methyl 3,4,5-Tris(p-alkoxybenzyloxy)benzoates and (15-Crown-5)methyl 3,4,5-Tris(p-dodecyloxy)benzoate. J. Chem. Soc. Perkin Trans..

[55] Zhou P, Blubaum JE, Bums CT, Natale NR (1997). The direct synthesis of 2-oxazolines from carboxyfic esters using lanthanide chloride as catalyst. Tetrahedron Lett..

[56] Ilkgul B, Gunes D, Sirkecioglu O, Bicak N (2010). Synthesis of 2-oxazolines via boron esters of N-(2-hydroxyethyl) amides. Tetrahedron Lett..

[57] Mohammadpoor-Baltork I, Moghadam M, Tangestaninejad S, Mirkhani V, Hojati SF (2008). Environmental-friendly synthesis of oxazolines, imidazolines and thiazolines catalyzed by tungstophosphoric acid. Catal. Commun..

[58] Bandgar BP, Pandit SS (2003). Direct synthesis of 2-oxazolines from carboxylic acids using 2-chloro-4,6-dimethoxy-1,3,5-triazine under mild conditions. Tetrahedron Lett..

[59] Kangani CO, Day BW (2009). A novel and direct synthesis of 1,3,4-oxadiazoles or oxazolines from carboxylic acids using cyanuric chloride/indium. Tetrahedron Lett..

[60] Verbraeken, B., Lava, K. & Hoogenboom, R. Poly(2-oxazoline)s. in *Encyclopedia of Polymer Science and Technology* (John Wiley & Sons, Inc., 2002).

[61] Huang H, Yang W, Chen Z, Lai Z, Sun J (2019). A mild catalytic synthesis of 2-oxazolines: via oxetane ring-opening: Rapid access to a diverse family of natural products. Chem. Sci..

[62] Holerca MN, Percec V (2000). ^1^H NMR spectroscopic investigation of the mechanism of 2- substituted-2-oxazoline ring formation and of the hydrolysis of the corresponding oxazolinium salts. Eur. J. Org. Chem..

[63] Xu Q, Li Z (2009). A facile synthesis of 2-oxazolines using a PPh3-DDQ system. Tetrahedron Lett..

[64] Kagiya T, Matsuda T (1972). Selective polymerization of 2-isopropenyl-2-oxazoline and cross-linking reaction of the polymers. Polym. J..

[65] Morimoto H, Fujiwara R, Shimizu Y, Morisaki K, Ohshima T (2014). Lanthanum(III) triflate catalyzed direct amidation of esters. Org. Lett..

[66] Pásztói B (2021). Quasiliving cationic ring-opening polymerization of 2-ethyl-2-oxazoline in benzotrifluoride, as an alternative reaction medium. Polymer.

