# Author Correction: Synthesis and characterization of dendritic compounds containing nitrogen: monomer precursors in the construction of biomimetic membranes

**DOI:** 10.1038/s41598-024-57172-1

**Published:** 2024-03-20

**Authors:** Jordi Guardià, Alireza Zare, John Eleeza, Marta Giamberini, José Antonio Reina, Xavier Montané

**Affiliations:** 1 Department of Analytical Chemistry and Organic Chemistry, Universitat Rovira i Virgili (URV), C/Marcel·lí Domingo 1, 43007 Tarragona, Spain; 2 Department of Chemical Engineering, Universitat Rovira i Virgili (URV), Av. Països Catalans 26, 43007 Tarragona, Spain

Correction to: *Scientific Reports* 10.1038/s41598-022-05747-1, published online 02 February 2022

The Acknowledgements section in the original version of this Article was incomplete.

“We are grateful for the financial support from the Ministerio de Ciencia e Innovación, grant number PID2020-116322RB-C32. This project has received funding from the European Union’s Horizon 2020 research and innovation programme under the Marie Skłodowska-Curie grant agreement No. 713679. Open access funding was provided by the 2021OPEN call of Universitat Rovira i Virgili (URV). The authors are grateful to Francesc Gispert (Servei de Recursos Científics i Tècnics, Universitat Rovira i Virgili) for his help in XRD experiments.”

now reads:

“We are grateful for the financial support from the Ministerio de Ciencia e Innovación, grant number PID2020-116322RB-C32. This project has received funding from the European Union’s Horizon 2020 research and innovation programme under the Marie Skłodowska-Curie grant agreement No. 713679. The authors also thank the Universitat Rovira i Virgili and Diputació de Tarragona for a pre-doctoral contract (2020PMF-PIPF-21) within its Martí i Franquès programme. Open access funding was provided by the 2021OPEN call of Universitat Rovira i Virgili (URV). The authors are grateful to Francesc Gispert (Servei de Recursos Científics i Tècnics, Universitat Rovira i Virgili) for his help in XRD experiments.”

The original Article has been corrected.

