# Supplementary material for: Synthesis and characterization of dendritic compounds containing nitrogen: monomer precursors in the construction of biomimetic membranes
Source: Sci Rep. 2022 Feb 2;12:1725. doi: 10.1038/s41598-022-05747-1 (PMC8810782; doi:10.1038/s41598-022-05747-1)
Supplement: Supplementary file 1 — Supplementary Information. [file 41598_2022_5747_MOESM1_ESM.pdf]

Supplementary Information: Synthesis and characterization of dendritic compounds containing nitrogen: monomer precursors in the construction of biomimetic membranes

Jordi Guardià[1], Alireza Zare[2], John Eleeza[1], José Antonio Reina[1], Marta Giamberini[2] & Xavier Montané[1,*]

[1] Universitat Rovira i Virgili (URV), Department of Analytical Chemistry and Organic Chemistry, C/Marcel·lí Domingo 1, 43007 Tarragona, Spain

[2] Universitat Rovira i Virgili (URV), Department of Chemical Engineering, Av. Països Catalans 26, 43007 Tarragona, Spain

* Corresponding authors: xavier.montane@urv.cat; Tel.: +34-977558288

CONTENTS OF THE SUPPLEMENTARY INFORMATION

1. **Experimental section** S3
2. **Supplementary figures of the synthetic approaches investigated** S5

Figure S1. Amidation of TAPAc with ethanolamine.

3. **Supplementary figures for NMR investigations** S6

Figure S2. COSY NMR spectra of TAPAm.

Figure S3. HSQC spectra of TAPAm. In the spectra, the blue signals correspond to -CH- and $-CH_3$ signals, while the red ones correspond to $-CH_2-$ signals.

Figure S4. COSY spectra of TAPOx.

Figure S5. HSQC spectra of TAPOx. In the spectra, the blue signals correspond to -CH- and $-CH_3$ signals, while the red ones correspond to $-CH_2-$ signals.

Figure S6. HMBC spectra of TAPOx.

4. **Supplementary figures for thermal and mesomorphic investigations** S11

Figure S7. XRD patterns of TAPOx recorded on heating at: (a) 70 °C and (b) 100 °C on first heating; and (c) 70 °C on second heating.

Figure S8. DTGA curves of (a) TAPAm, (b) TAPOx and (c) TAPEs recorded at a heating rate of 10 °C/min in nitrogen atmosphere.

1. Experimental section

Synthesis of *N*-(2-hydroxyethyl)-3,4,5-tris(4-dodecyloxybenzyloxy)benzamide (TAPAm)

Direct amidation method using DCC as coupling agent. In a round-bottomed flask, 1.01 g of TAPAc (1.01 mmol) was dissolved into 10 mL of chloroform. The solution was stirred at 0 °C in an ice-water bath for 15 min. Then, DMAP (102.2 mg, 0.83 mmol) was added, and the solution was kept under stirring at 0 °C during 15 min more. After that, DCC (210.9 mg, 1.02 mmol) was added, leaving the magnetic stirring for additionally 15 min at the same temperature. At this point, ethanolamine (0.18 mL, 2.98 mmol) was added dropwise. When the addition of ethanolamine was completed, the reaction mixture was warmed to room temperature and kept under stirring for 4 days, during which it was monitored by TLC using n-hexane/ethyl acetate (1:2) as mixture of eluents (A complete conversion of TAPAc was detected after 4 days of reaction). At this time, the crude of the reaction was filtered to eliminate the undesirable N, N'-dicyclohexylurea and the solvent was eliminated under vacuum at the rotavapor. The resulting orange solid was purified by flash column chromatography using a gradient of n-hexane/ethyl acetate (starting proportion = 2:1) as mixture of eluents. Nevertheless, a mixture of TAPAm and N,N'-dicyclohexylurea was always isolated.

Synthesis of 2-(3,4,5-tris(4-dodecyloxybenzyloxy)phenyl)-2-oxazoline (TAPOx)

Direct synthesis of TAPOx from TAPAm using thionyl chloride ($SOCl_2$) and DBU. In a round bottomed flask, 400.5 mg of TAPAm (0.38 mmol) were dissolved in 5 mL of DCM at room temperature. After that, DBU (346.3 mg, 2.31 mmol) was added, which was followed by the dropwise addition of thionyl chloride (147.4 mg, 1.15 mmol). The reaction was monitored by TLC using n-hexane/ethyl acetate (1:2) as mixture of eluents. After complete conversion of TAPAm (2 h), the formation of TAPOx was not detected by ^{1}H NMR in the crude mixture.

Direct synthesis of TAPOx from TAPEs using Lanthanum (III) salts as catalyst. Anhydrous Lanthanum (III) chloride ($LaCl_3$) (5, 10, 20 % mol respect to TAPEs) or anhydrous Lanthanum (III) triflate ($LaTf_3$) (10 % mol regarding TAPEs) and ethanolamine (0.15 mL, 2.48 mmol) were dissolved in 10 mL of dry toluene inside a two neck round bottom flask under argon flow conditions. Subsequently, n-BuLi (0.90 mL, 2.25 mmol) was added dropwise at 0 °C, keeping the mixture under stirring for 15 min. Then, the reaction mixture was warmed to reflux at 100 °C, when TAPEs (1.01 g, 0.99 mmol) was added carefully. The reaction was monitored by TLC using n-hexane/ethyl acetate (1:2) as mixture of eluents. When a total conversion of TAPEs was achieved (24 h), the crude mixture was cooled to RT and washed several times with water. Finally, the organic layer was dried over anhydrous $MgSO_4$, and the solvent was vacuum evaporated. After that, the obtained solid was purified by flash column chromatography using n-hexane/ethyl acetate (2:1) as mixture of eluents. Nonetheless, TAPOx was not isolated in any of the collected fractions.

Preliminary polymerization studies

Polymerization of TAPOx with toluene as solvent. In a previously flame-dried Schlenk tube, 500.0 mg of TAPOx monomer (0.49 mmol) were dissolved in 5.0 mL of dry toluene. Then, 1 % of methyl tosylate were added at room temperature. After that, the tube was immersed into a preheated oil bath at 130 °C and kept under stirring in an argon flow

conditions for 12 days. The reaction was monitored by ^{1}H NMR. However, only unreacted TAPOx monomer was detected.

Polymerization of TAPOx with benzotrifluoride as solvent. In a previously flame-dried Schlenk tube, 503.0 mg of TAPOx monomer (0.49 mmol) were dissolved in 2.5 mL of anhydrous benzotrifluoride. Then, 1 % of methyl tosylate were added at room temperature. After that, the tube was immersed into a preheated oil bath at 90 °C and kept under stirring in an argon flow conditions for 10 days. The reaction was monitored by ^{1}H NMR. Nevertheless, only unreacted TAPOx monomer was detected after 10 days of reaction.

2. Complementary figures of the synthetic approaches investigated

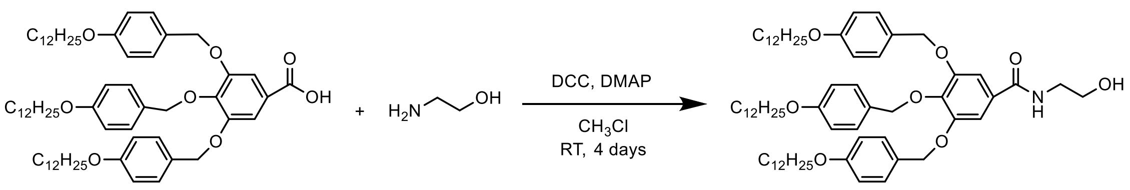

Figure S1. Amidation of TAPAc with ethanolamine.

3. Complementary figures for NMR investigations

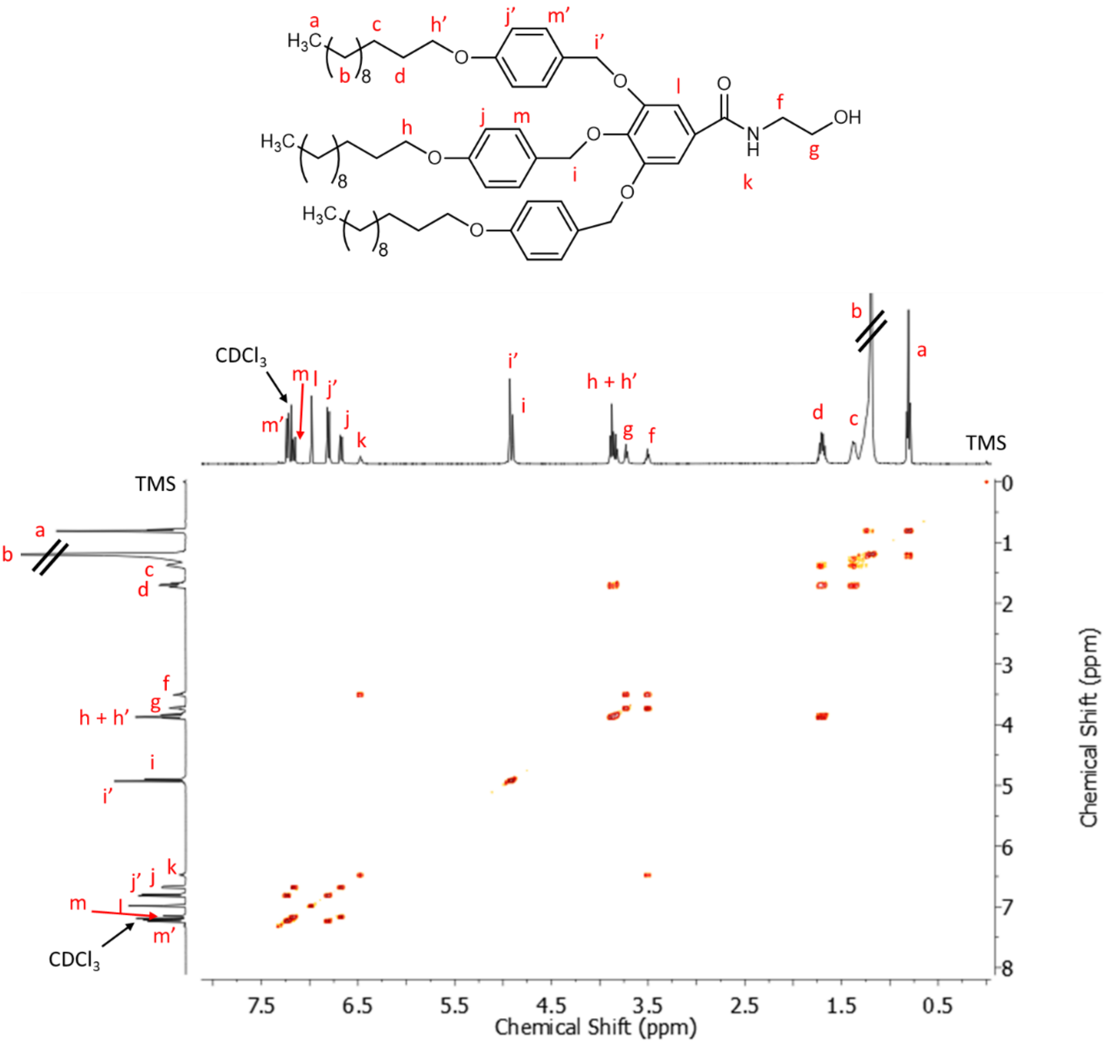

Figure S2. COSY NMR spectra of TAPAm.

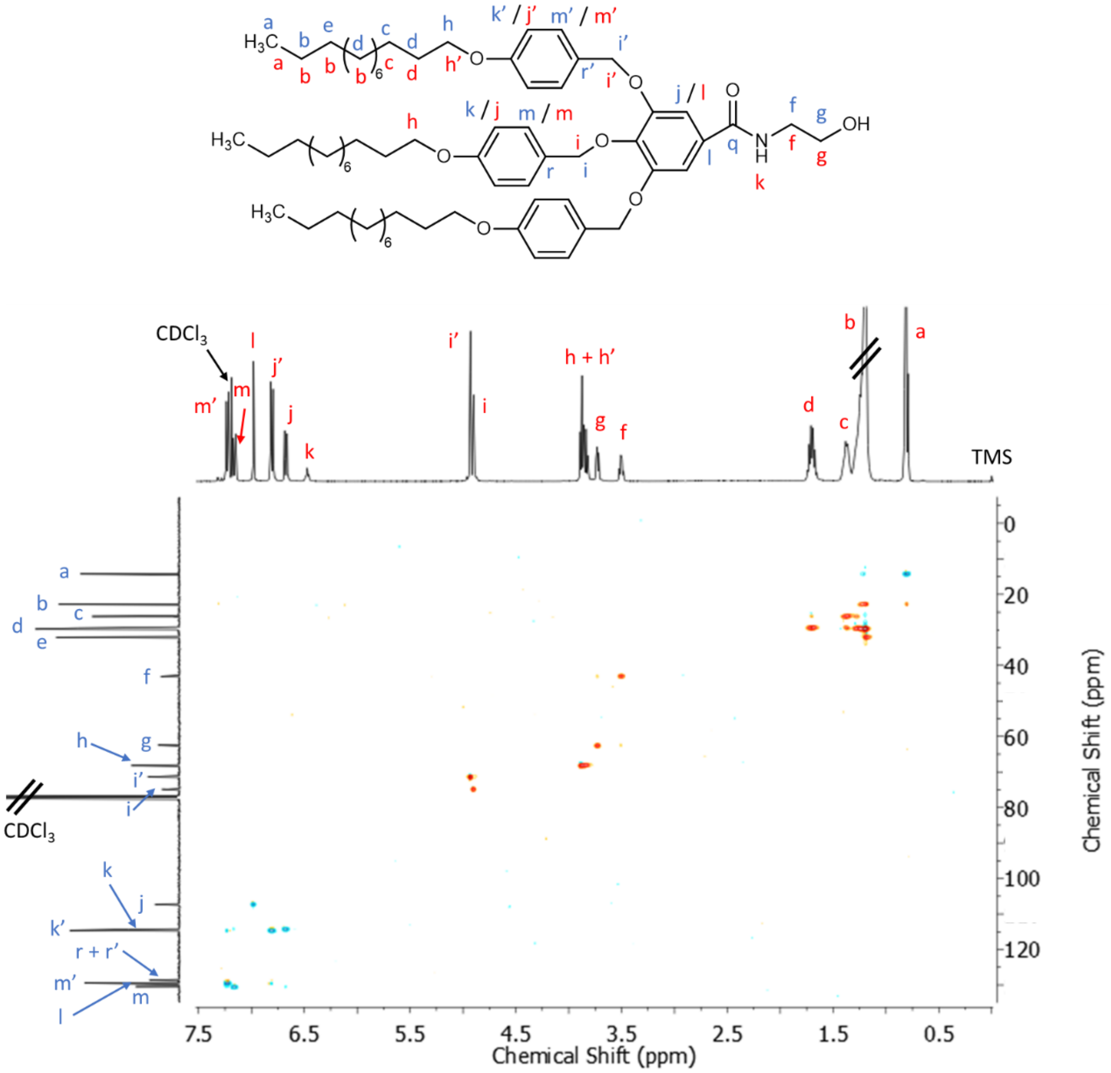

Figure S3. HSQC spectra of TAPAm. In the spectra, the blue signals correspond to -CH- and $-CH_3$ signals, while the red ones correspond to $-CH_2-$ signals.

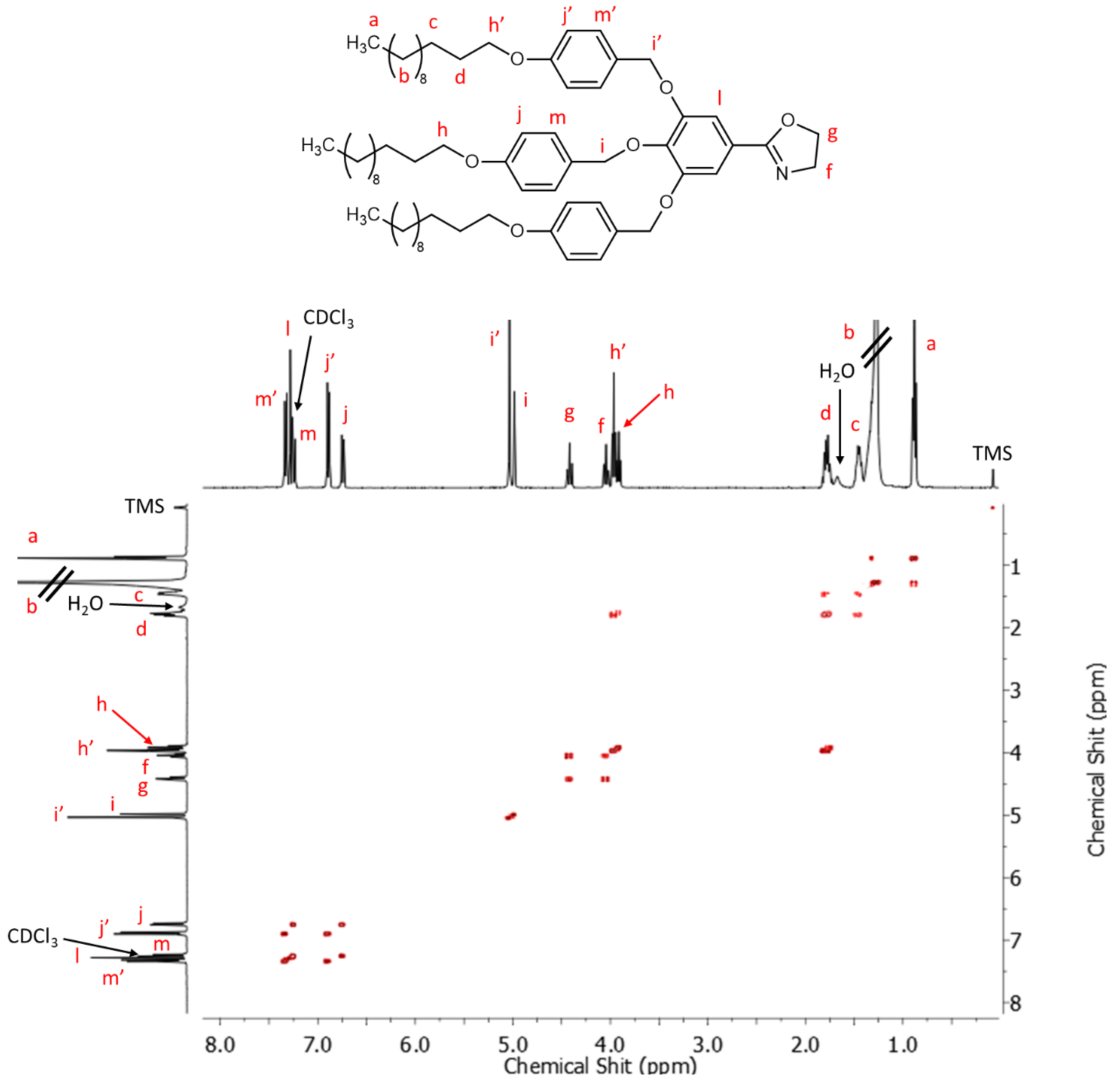

Figure S4. COSY spectra of TAPOx.

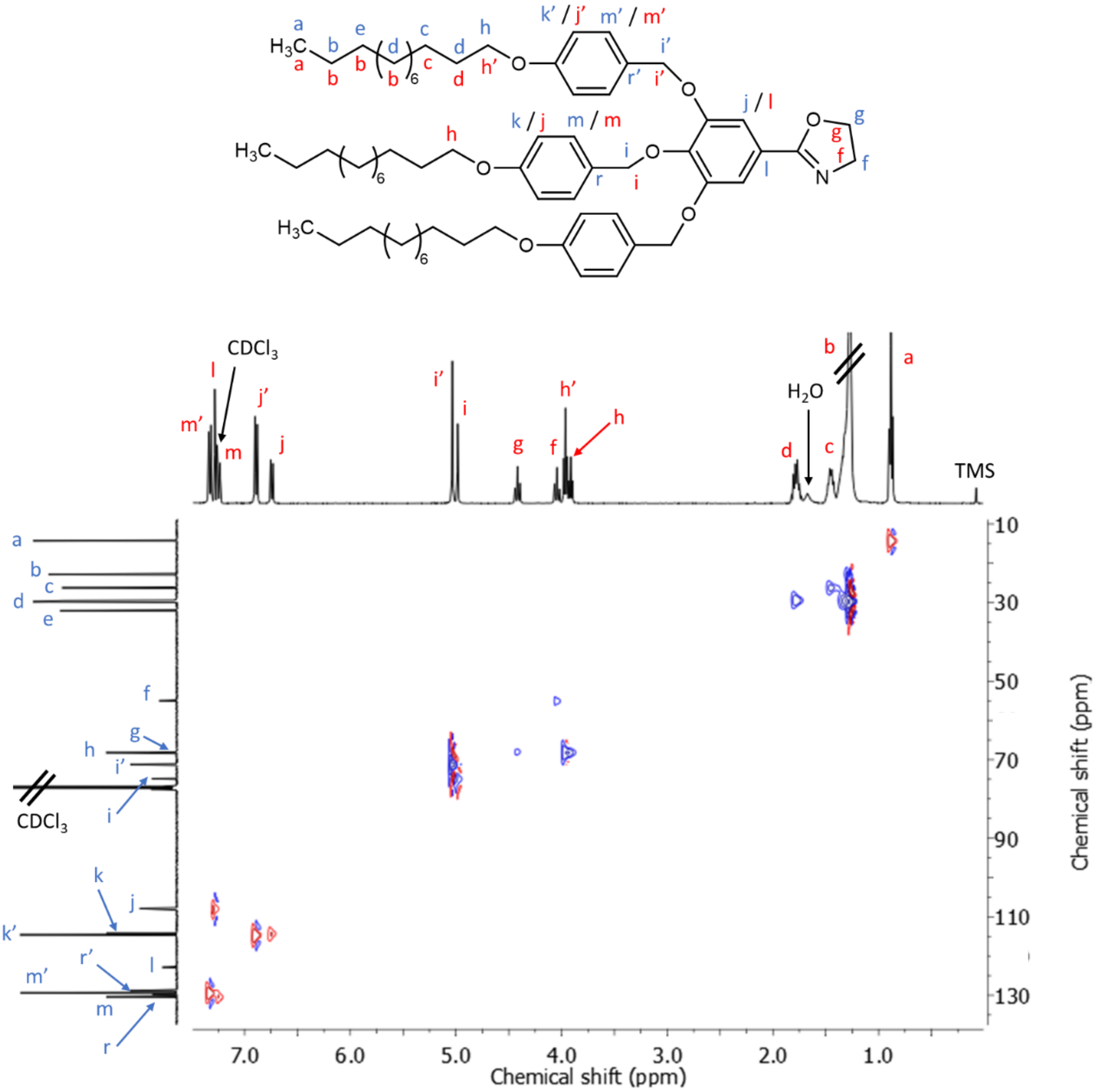

Figure S5. HSQC spectra of TAPOx. In the spectra, the blue signals correspond to -CH- and -CH_3 signals, while the red ones correspond to -CH_2- signals.

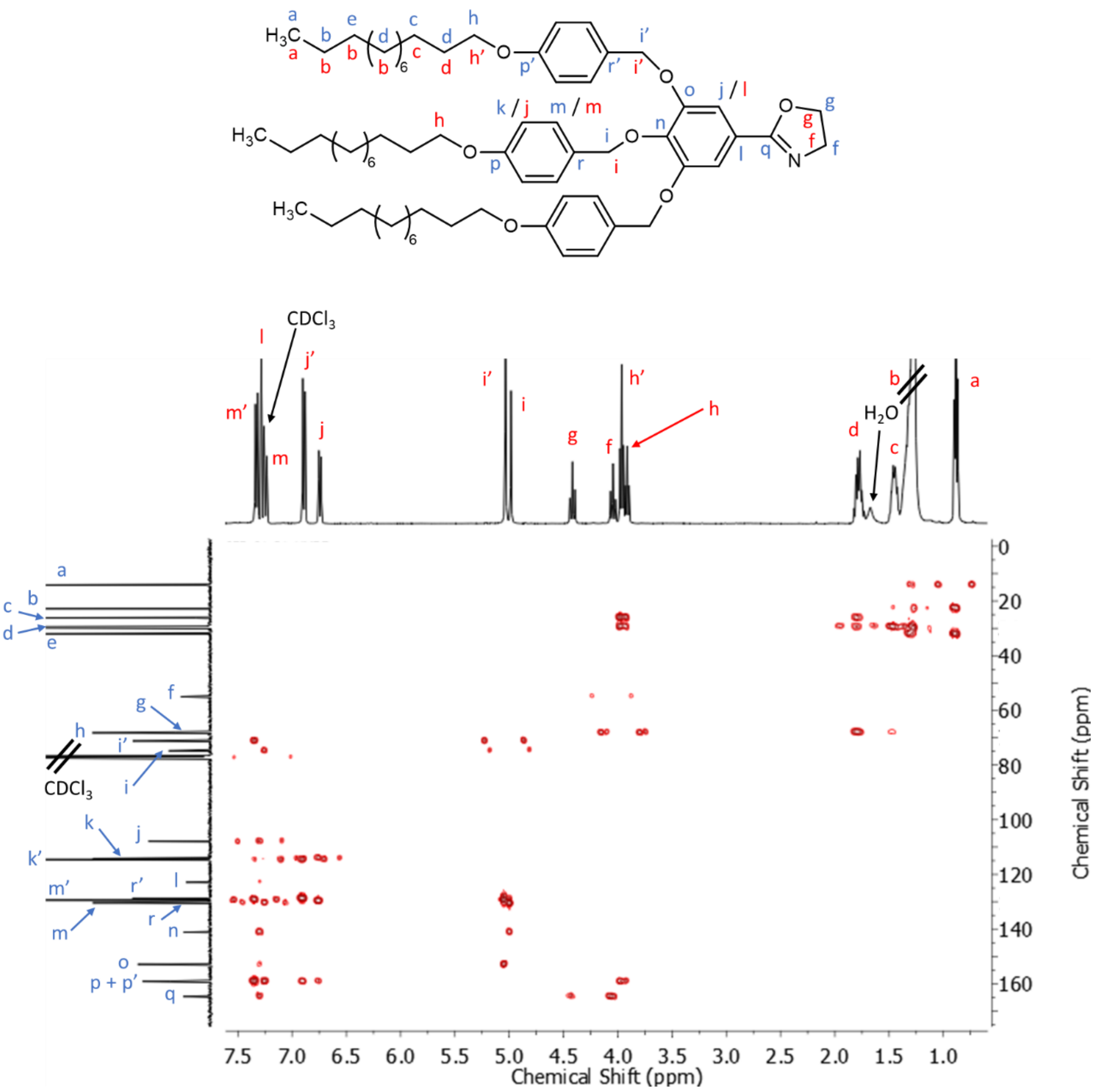

Figure S6. HMBC spectra of TAPOx.

4. Supplementary figures for thermal and mesomorphic investigations

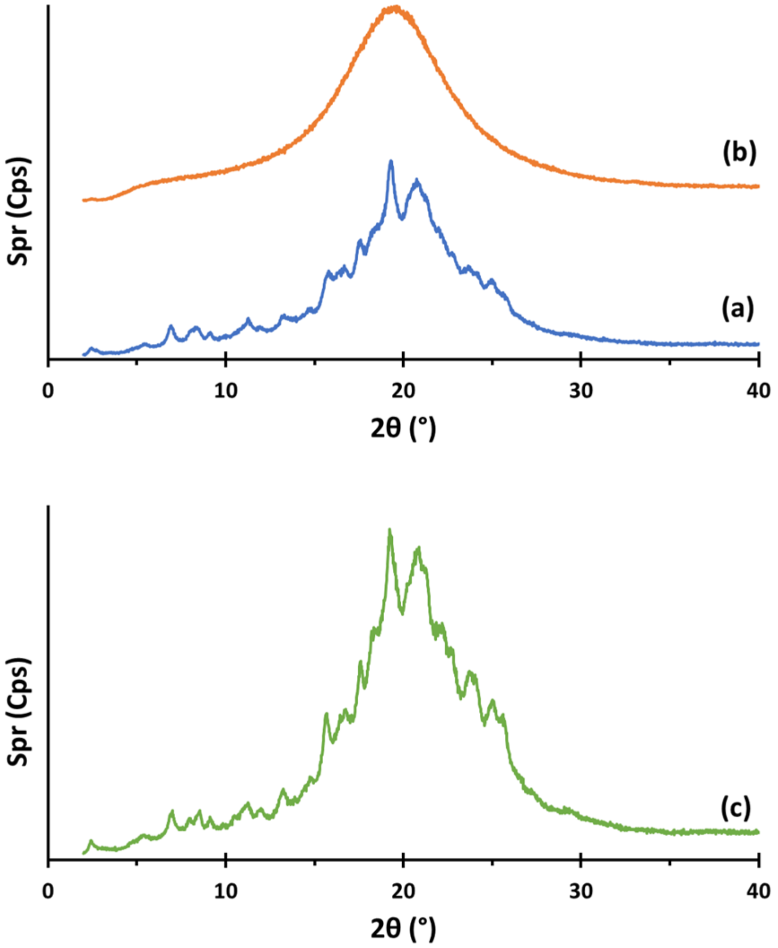

Figure S7. XRD patterns of TAPOx recorded on heating at: (a) 70 °C and (b) 100 °C on first heating; and (c) 70 °C on second heating.

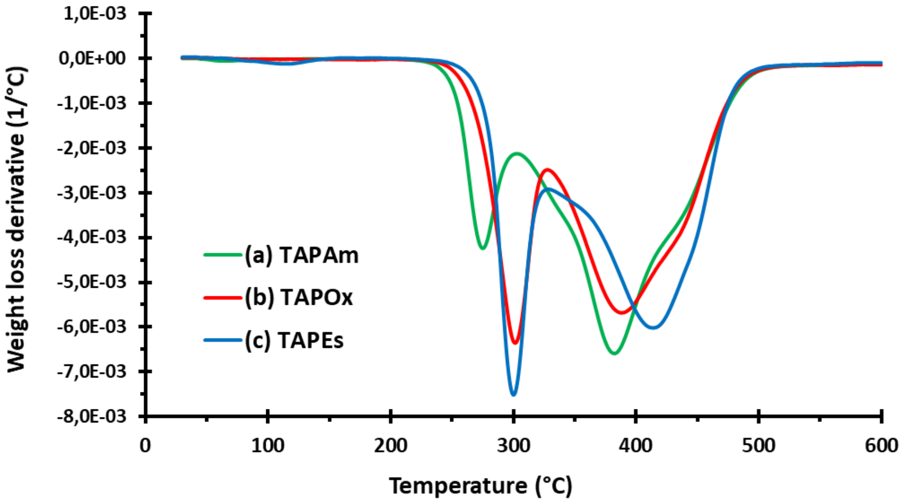

Figure S8. DTGA curves of (a) TAPAm, (b) TAPOx and (c) TAPEs recorded at a heating rate of 10 °C/min in nitrogen atmosphere.
